# An overview and evaluation of first-trimester physiological fetal human anatomy using 3-dimensional ultrasound combined with virtual reality techniques

**DOI:** 10.1093/humrep/deaf112

**Published:** 2025-06-27

**Authors:** Kristel Zandbergen, Annemarie G M G J Mulders, Carsten S Pietersma, Anton H J Koning, Bernadette S de Bakker, Eric A P Steegers, Melek Rousian

**Affiliations:** Department of Obstetrics and Gynecology, Erasmus MC, University Medical Center, Rotterdam, The Netherlands; Department of Obstetrics and Gynecology, Erasmus MC, University Medical Center, Rotterdam, The Netherlands; Department of Obstetrics and Gynecology, Erasmus MC, University Medical Center, Rotterdam, The Netherlands; Department of Pathology, Erasmus MC, University Medical Center, Rotterdam, The Netherlands; Department of Obstetrics and Gynecology, Amsterdam UMC Location University of Amsterdam, Amsterdam, The Netherlands; Amsterdam Reproduction and Development Research Institute, Amsterdam, The Netherlands; Department of Pediatric Surgery, Sophia Children’s Hospital, University Medical Center Rotterdam, Rotterdam, The Netherlands; Department of Obstetrics and Gynecology, Erasmus MC, University Medical Center, Rotterdam, The Netherlands; Department of Obstetrics and Gynecology, Erasmus MC, University Medical Center, Rotterdam, The Netherlands

**Keywords:** virtual reality, 3-dimensional ultrasound, first-trimester, fetal human anatomy, 2-dimensional ultrasound

## Abstract

**STUDY QUESTION:**

What (physiological) first-trimester fetal anatomic structures can be discerned by ultrasound (US) and can these structures be visualized using 3-dimensional (3D) US combined with virtual reality (VR) in a prospective clinical setting?

**SUMMARY ANSWER:**

3D US combined with VR techniques has shown to be applicable for the assessment of fetal anatomy in the first trimester and may serve as a valuable tool for both professional training and patient counseling.

**WHAT IS KNOWN ALREADY:**

Due to technological developments, new imaging modalities are becoming available and the visualization of fetal anatomic structures continues to improve. Consequently, in recent years, the focus of antenatal US screening has progressively shifted toward the first trimester of pregnancy. To further assess the applicability of new imaging techniques in detecting anomalies it is essential first to demonstrate the visibility of physiological fetal anatomy. Until today, an extended overview of first-trimester physiological fetal structures discernable by US is missing and most knowledge on first-trimester fetal anatomy is still based on imaging modalities other than US evaluating *ex vivo* human subjects.

**STUDY DESIGN, SIZE, DURATION:**

A systematic literature search was performed by two independent reviewers in five electronic databases. All studies published between January 1946 and January 2024 in the English language, assessing ultrasonically discernible fetal structures in the first trimester of pregnancy were included. Subsequent, a literature-based checklist of ultrasonically discernible first-trimester fetal structures was developed. According to the constructed checklist, an offline VR assessment of 3D and 4-dimensional (4D) US datasets for discernable fetal structures was conducted.

**PARTICIPANTS/MATERIALS, SETTING, METHODS:**

In 55 high-risk pregnancies between a gestational age (GA) of 11^+0^–13^+6 ^weeks 3D and 4D US datasets were collected prospectively and selected based on their quality. The US datasets were offline evaluated for discernable fetal structures as indicated on the predetermined checklist using VR by two trained observers. After offline VR assessment, visibility rates for all ultrasonically discernible structures were calculated as a proportion of the total number of US datasets.

**MAIN RESULTS AND THE ROLE OF CHANCE:**

A systematic literature search (N = 15 874 studies retrieved) resulted in the inclusion and quality assessment of 372 studies, from which 81 ultrasonically discernible fetal structures were identified and incorporated into the checklist. An offline VR assessment was performed in 3D and 4D US datasets of 55 pregnancies with a mean GA of 12 + 6 weeks (SD 0.4 days). The mean visibility rate of all fetal structures incorporated in the checklist was 82.2%.

**LIMITATIONS, REASONS FOR CAUTION:**

A key limitation of this study is the lack of targeted US examination during the acquisition of all 3D and 4D US datasets. A targeted approach could improve dataset quality and visibility rates in the offline evaluation of fetal anatomy using 3D US and VR. Additionally, the selection of high-quality 3D US datasets may introduce selection bias, which could impact the generalizability of the findings. Furthermore, since the study population was recruited from a tertiary referral center where US examinations were performed by experienced sonographers using a high-frequency transvaginal US transducer, there may be limitations in extrapolating these results to the broader general population, where access to such specialized expertise and equipment may be more limited.

**WIDER IMPLICATIONS OF THE FINDINGS:**

This is the first comprehensive literature-based overview of first-trimester physiological fetal structures discernable by US, which has been evaluated in a prospective clinical setting. Moreover, this study underlines the potential added value of the use of 3D US combined with VR, both as educational and reference resource for professionals and counseling in daily clinical practice.

**STUDY FUNDING/COMPETING INTEREST(S):**

No specific funding was used for the execution of this study. Departmental funds were utilized to support the authors throughout the study period and during manuscript preparation. These funds were provided by the Department of Obstetrics and Gynaecology at the Erasmus MC University Medical Center, Rotterdam, The Netherlands. The authors declare no conflicts of interest.

**TRIAL REGISTRATION NUMBER:**

N/A.

## Introduction

Due to technical advances in ultrasound (US), the emphasis of antenatal US screening is moving toward the first trimester of pregnancy. Currently, 32–61% of congenital anomalies in the first trimester can be detected by 2-dimensional (2D) US (Karim *et al.*, 2017; Syngelaki *et al.*, 2019). In part as a result of the practice guidelines set by the International Society of Ultrasound in Obstetrics and Gynecology (ISUOG), the assessment of fetal anatomy is increasingly being implemented as a key objective of first-trimester US screening (Bronsgeest *et al.*, 2023; International Society of Ultrasound in Obstetrics and Gynecology *et al.*, 2023). To further assess the applicability of new imaging techniques in the detection of anomalies it is essential first to demonstrate the visibility of physiological fetal anatomy. Until today, an extended overview of first-trimester physiological fetal structures discernable by US is missing and most knowledge on first-trimester fetal anatomy is still based on imaging modalities other than US evaluating *ex vivo* human subjects (Yamada *et al.*, 2010; Pooh *et al.*, 2011; de Bakker *et al.*, 2016). Since the introduction of 3-dimensional (3D) US, this technique is typically utilized as a complementary diagnostic tool in addition to 2D US. Nevertheless, a major shortcoming of assessing 3D US datasets on a 2D screen is the lack of inclusion of information on the third dimension, namely depth. To fully benefit from all three dimensions offered by 3D US datasets, the Erasmus MC has been developing and successfully utilizing an advanced imaging technique known as virtual reality (VR) for over a decade, both for research and clinical purposes (Rousian *et al.*, 2011; Baken *et al.*, 2014; Pietersma *et al.*, 2020). The use of an in-house developed volume rendering application called V-Scope allows displaying 3D US data as holograms. In addition, VR facilitates unlimited magnification and rotation of the acquired 3D US datasets around all three axes and could thus serve as a valuable *in vivo* instrument for the assessment of fetal anatomy and counseling or training tool. To evaluate the applicability of this technique, it is important to consider its effectiveness in identifying visible first-trimester physiological fetal structures as reported in the literature.

Therefore, our first aim is to create a comprehensive overview of physiological fetal anatomy discernable by US in the first trimester of pregnancy. Second, we aim to evaluate prospectively the applicability of 3D US combined with VR in the visualization of first-trimester fetal anatomy.

## Materials and methods

The first part of this study consists of a systematic literature review to construct a comprehensive overview of physiological fetal anatomy discernable by US in the first trimester of pregnancy.

The second part consists of an offline VR assessment according to the constructed checklist using prospectively acquired 3D US datasets in 55 first-trimester pregnancies (between 11^+0^ and 13^+6^ weeks’ gestation).

### Search methods

A search in Embase, Medline, Web of Science, Cochrane Central Register of Controlled Trials (CENTRAL), and Google Scholar was conducted. Our search strategy included keywords related to the use of US imaging and the first trimester of pregnancy, without limiting the focus to the 11^+0^–13^+6 ^week scan, but including US in general. These keywords were combined with search terms describing either fetal anatomy or discernible fetal structures (Supplementary Table S1). A protocol of this systematic review has been registered in PROSPERO International prospective register of systematic reviews (2020: CRD42020215805, available from: http://www.crd.york.ac.uk/PROSPERO/display_record.asp?ID=CRD42020215805).

Studies published between January 1946 and January 2024 in English language were included. After de-duplication, studies eligible for inclusion had to describe any discernible fetal structures in the first trimester of pregnancy (explicitly described ≤13^+6^ weeks’ gestation) using 2D, 3D, or 4-dimensional (4D) US technology. Studies reporting on soft markers (fetal sonographic findings that are generally not considered as anomalies but are indicative of an increased age-adjusted risk of an underlying fetal aneuploidy or some non-chromosomal abnormalities), or biometric measurements were excluded. Studies reporting on structures that are only discernable during a specific period, due to the process of physiological embryonic development (e.g. rhombencephalon as a preliminary stage of the pons) were not included in the constructed checklist. Also, ultrasonically discernible fetal structures visible as a result of pathology or dynamic features (e.g. color Doppler imaging (CDI)) were excluded, except for cardiac structures and the number of umbilical cord vessels. Additionally, letters to the editor, editorials, conference abstracts, and narrative reviews were excluded. Reviewers (G.B. and K.Z.) independently screened the titles and abstracts of all studies. Any disagreement about the inclusion or exclusion of an article was resolved through discussion with a third reviewer (A.M.).

### Quality assessment

The quality of the included articles was assessed utilizing the Newcastle-Ottawa Quality Assessment Scale (NOS). The assessment of methodological quality was performed by one reviewer (K.Z.), following the NOS for cohort and case–control studies (Supplementary Data File S1) and the NOS adapted for Cross-Sectional studies (Supplementary Data File S2), that contemplates three categories (Selection, Comparability, and Outcomes/Exposure) relating to methodological quality. Studies with Newcastle-Ottawa form scores of ≥7 were considered as high-quality, 5–6 as moderate quality and 0–4 as low-quality studies (Stang, 2010).

### Checklist construction

First, baseline characteristics of all included studies were extracted. This included title, author, year of publication, study design, and population characteristics (i.e. a high- or low-risk pregnancy for an underlying fetal aneuploidy or non-chromosomal abnormality) as reported in the article. Then, for every included study, the ultrasonically discernible fetal structures were documented in a Microsoft Excel overview and categorized according to their function and/or location. If available, the following data for the corresponding structure was retrieved: US approach (transabdominal (TA) or transvaginal (TV)), US technology (2D, 3D, or 4D US), crown-rump length (CRL), and gestational age (GA). In case GA was not reported, an estimation equation based on the CRL was used to calculate the GA (Robinson and Fleming, 1975). Further, as a product of the literature review, a checklist of ultrasonically discernible fetal structures in the first trimester of pregnancy was constructed.

### Data acquirement

For the second part of the study, we focused on the assessment of prospectively collected 3D and 4D US datasets in 55 first-trimester pregnancies (between 11^+0^–13^+6^ weeks’ gestation) from a high-risk population (Pietersma *et al.*, 2020). High-risk was defined as, or in case of: (i) fetus with one first-degree relative with structural anomaly not based on a genome abnormality, (ii) a fetus with two second-degree or other, further relatives with a similar anomaly, (iii) a maternal disease (e.g. diabetes mellitus), (iv) the pregnancy was conceived after ICSI, (v) substance use of teratogenic medication/drugs, or and (vi) monozygotic multiple pregnancy.

US examinations were performed by experienced sonographers on a Voluson E8 or E10 (GE Healthcare, Zipf, Austria) machine using a 4–9 MHz or 6–13 MHz high-frequency TV transducer or a 2–6 MHz TA transducer. Total scanning time was kept as low as possible (ALARA principle) according to international guidelines on safety of US in the first trimester of pregnancy (ter Haar, 2010). During an extensive structural 2D US examination and evaluation of all organ systems, 3D US datasets from the fetus were acquired and stored for offline assessment. In addition to the 3D US datasets, 4D spatio-temporal image correlation (STIC) datasets in B-mode and with CDI were acquired for assessment of the cardiovascular system and number of umbilical cord vessels.

Per pregnancy, the quality of the acquired and stored 3D and 4D STIC datasets was assessed according to a scoring system based on the presence of motion artifacts, shadowing artifacts, volume completeness, and blurriness. This allowed a total score of 0–4. US datasets with a score below 2 were considered low quality, those with a score of 3 were considered average quality, and those with a score of 4 were considered high quality. For the offline assessment, only 3D US datasets of average- (3) and high (4) quality were selected. The quality of 3D US datasets was given priority over the quality of the 4D STIC datasets. Consequently, the 4D STIC datasets from the corresponding pregnancy could be of a lower quality score.

The extensive VR FETUS study design has previously been published (Pietersma *et al.*, 2020). Hospital or midwifery charts were obtained to complete follow-up on all participants. In our cohort, only datasets from fetuses without congenital anomalies were selected.

### Ethical approval

The study protocol was approved by the Erasmus MC Institutional Review Board (MEC-2004-227) and all participating women and their partners signed written informed consent at enrolment (reference number: NL58563.078.16).

### Offline assessment using 3D US combined with VR

Offline VR assessment using the V-Scope software was performed within the I-Space, which is an immersive 4-walled VR system, available at the Erasmus Medical Center. The V-Scope volume rendering application developed at the Erasmus Medical Center is used to create an interactive ‘hologram’ of the 3D US dataset (Koning *et al.*, 2009). This hologram can be manipulated by using a virtual pointer that is controlled by a wireless joystick, facilitating assessment of fetal structures from multiple angles and planes as demonstrated in Video 1. Further demonstration of the clinical application of 3D VR is provided in Supplementary Video S1. Structures identified following the VR assessment, not included in our literature search (because they were not explicitly described before 13^+6^ weeks’ gestation) were verified in micro-CT scan of histologically sectioned human fetuses from the Dutch Fetal Biobank. These structures were then incorporated in the earlier constructed checklist and evaluated in the remaining 3D US datasets. After an offline VR analysis by two trained observers (G.B. and K.Z.) according to the constructed literature-based checklist, visibility rates for all ultrasonically discernible structures were calculated as a proportion of the total number of US datasets.

## Results

### Literature search

**Figure 1.**
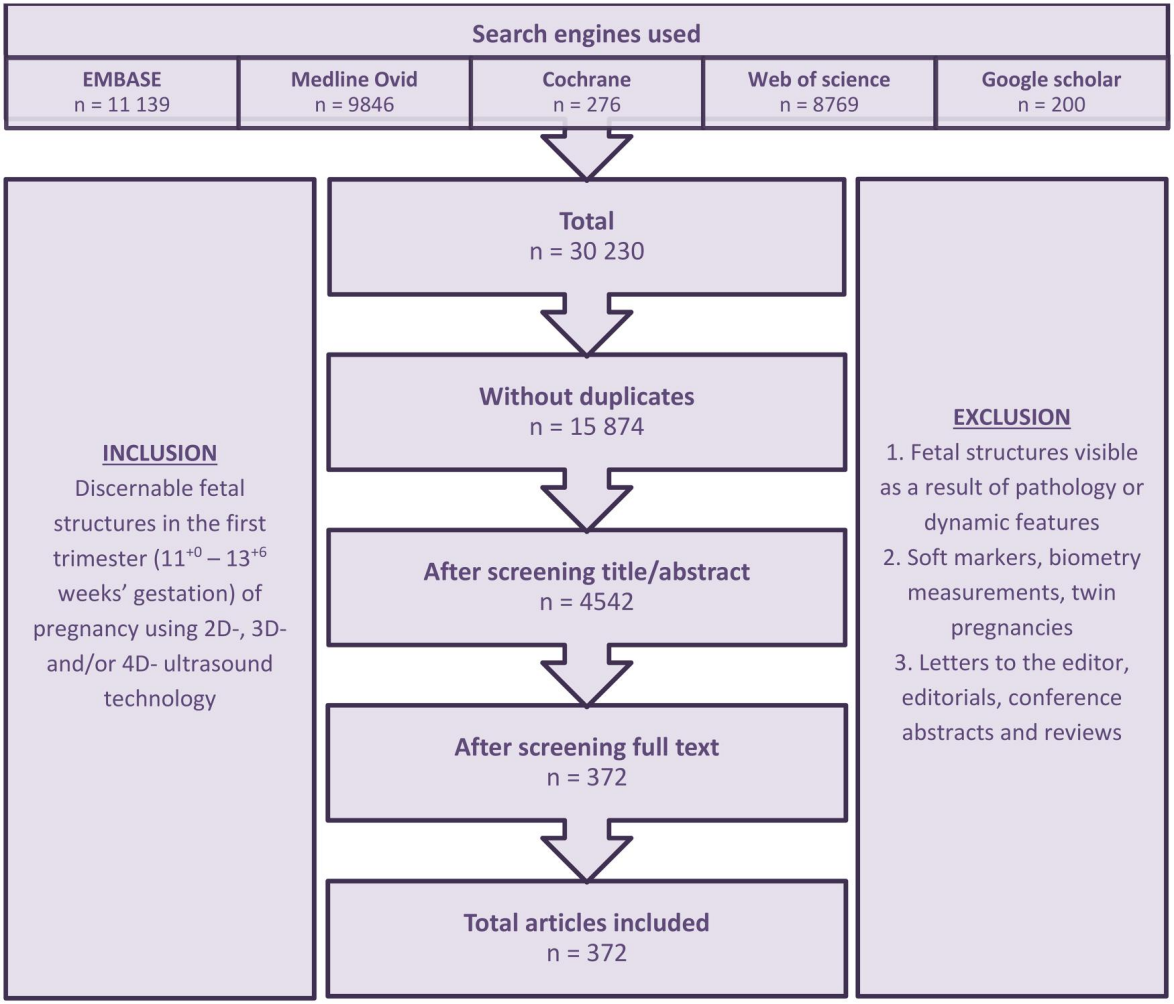
**Selection process of literature review and number of included articles**.

**Figure 2.**
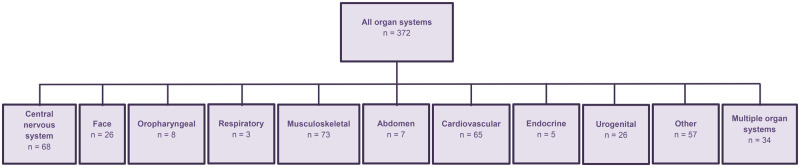
**The included studies categorized into subgroups based on the function and/or location of the described structures**.

Figure 1 shows the flowchart of the selection process of studies describing first-trimester physiological fetal anatomy discernable by US. Figure 2 categorizes the included studies into subgroups based on the function and/or location of the described structure.

The initial search strategy yielded 30 230 studies of which 15 874 unique studies remained after exclusion of duplicates. Screening of titles and abstracts led to a further exclusion of 11 332 studies not fulfilling the predefined eligibility criteria. The full texts of the remaining 4542 studies were screened, resulting in a total of 372 studies describing ultrasonically discernable fetal structures during the first trimester of pregnancy.

Table 1 provides an overview of the background characteristics of the included studies. The studies reported a low-risk population in 14.5% (n = 54), a high-risk population in 17.7% (n = 66), and a mixed population in 5.9% n = 22) in relation to an underlying fetal aneuploidy or non-chromosomal abnormality. In 61.8% (n = 230) of the included studies, the background of the study population was not reported. In 277 (74.5%) studies, a prospective study approach was used, whereas in 53 (14.2%) studies a retrospective design was used. Five (1.3%) studies mentioned a mixed (prospective and retrospective) design, 32 (8.6%) studies described a cross-sectional design, and 5 (1.3%) studies represented case reports. Regarding the US technology, 253 (68.0%) of the included studies used 2D US, with respectively 99 (26.6%) and 13 (3.5%) studies reporting on 3D and/or 4D US. In only seven (1.9%) of the included studies, 3D US was combined with VR technology. With regards to the US approach, in 134 (36.0%) studies US examinations were performed TA, in 87 (23.4%) studies TV and in 104 (28.0%) studies both TA and TV approaches were used. The remaining 47 (12.6%) studies did not report on the used US approach. In the included studies, GA of the performed US examinations ranged from 4^+1^ to 13^+6 ^weeks, with the majority (51.4%) of the examinations performed between 11^+0^ and 13^+6^ weeks’ gestation. Supplementary Table S2 provides an overview of included studies, classified per subgroup according to the function and/or location of the described structure (list of references included).

**Table 1.** Background characteristics of studies included in the constructed checklist (n = 372).

	n (%)
Study population	
Low-risk	54 (14.5)
High-risk	66 (17.7)
Mixed	22 (5.9)
Not reported	230 (61.8)
Study design	
Prospective	277 (74.5)
Retrospective	53 (14.2)
Prospective and retrospective	5 (1.3)
Cross-sectional	32 (8.6)
Case report	5 (1.3)
Ultrasound technology	
Two-dimensional (2D)	253 (68.0)
Three-dimensional (3D)	99 (26.6)
Four-dimensional (4D)	13 (3.5)
Three-dimensional combined with virtual reality (3D VR)	7 (1.9)
Ultrasound approach	
Transabdominal	134 (36.0)
Transvaginal	87 (23.4)
Both	104 (28.0)
Not reported	47 (12.6)

### Quality assessment

Based on the NOS, 34.5% (129) of the included studies scored as high quality, 55.9% (209) as moderate quality, and 9.6% (36) as low quality. These findings indicate the majority of the included studies were of moderate quality, with a notable proportion meeting the criteria for high quality.

### Checklist construction

A checklist of 81 ultrasonically discernible fetal structures in the first trimester of pregnancy (between 11^+0^ and 13^+6^ weeks’ gestation) was constructed based on our literature search (see column 1 of Table 2). Three fetal structures (main bronchus, uvula, and epiglottis) – not included in our literature search – were additionally identified during offline VR assessment. To confirm these findings, micro-CT scans of histologically sectioned human fetus from the Dutch Fetal Biobank were evaluated for the presence of these structures (Supplementary Fig. S1). The three identified structures (indicated with an * in Table 2) were incorporated in the constructed checklist and (re-)evaluated in all US datasets.

**Table 2.** Constructed checklist of first-trimester ultrasonically discernible fetal structures classified by subgroup including visibility rates displayed in percentage per structure (as a proportion of the total number of US datasets).

	Structure	Visibility rate n (%)
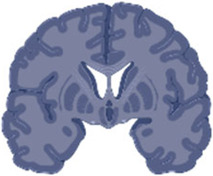 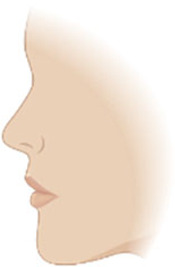 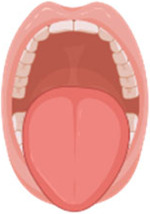 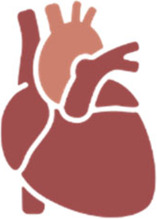 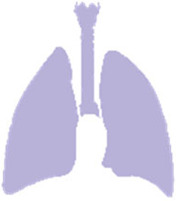 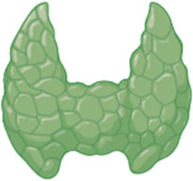 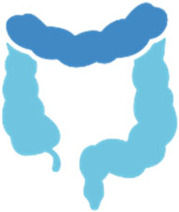 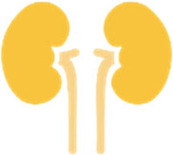 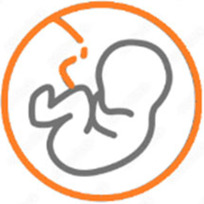 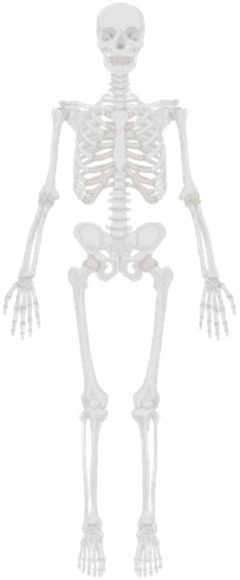	Central nervous system	
	Falx cerebri	55 (100)
	Choroid plexus	55 (100)
	Lateral ventricles	51 (92.7)
	Thalamus	50 (90.9)
	Cerebellum	54 (98.2)
	Cisterna magna	53 (96.4)
	Vermis	40 (72.7)
	Anterior membranous area	4 (7.3)
	Choroid plexus of 4th ventricle	42 (76.4)
	4th ventricle	53 (96.4)
	Sylvian aqueduct	50 (90.9)
	3rd ventricle	54 (98.2)
	Brain stem	35 (63.6)
	Midbrain	15 (27.3)
	Pons	43 (78.2)
	Medulla oblongata	40 (72.7)
	Subarachnoid space	48 (87.3)
	Sylvian fissure	53 (96.4)
	Face	
	Lens	55 (100)
	Vitreous	55 (100)
	Lips	49 (89.1)
	Tympanic ring	22 (40.0)
	Auricle	55 (100)
	Oropharyngeal	
	Uvula*	13 (23.6)
	Choans	52 (94.5)
	Palate	55 (100)
	Pharynx	55 (100)
	Larynx	48 (87.3)
	Epiglottis*	13 (23.6)
	Cardiovascular	
	Atria	48 (87.3)
	Ventricles	50 (90.9)
	Ventricle septum	47 (85.5)
	Aortic valve	31 (56.4)
	Aorta	44 (80.0)
	Pulmonary valve	26 (47.3)
	Pulmonary artery	38 (69.1)
	Mitral valve	47 (85.5)
	Tricuspid valve	47 (85.5)
	Vena cava inferior	17 (30.9)
	Vena cava superior	28 (50.9)
	Pulmonary veins	10 (18.2)
	Ductus arteriosus	25 (45.5)
	Respiratory	
	Trachea	46 (83.6)
	Main bronchus*	22 (40.0)
	Lungs	55 (100)
	Endocrine glands	
	Thyroid	53 (96.4)
	Thymus	42 (76.4)
	Adrenal glands	20 (36.4)
	Abdomen	
	Esophagus	5 (9.1)
	Stomach	55 (100)
	Bowel	55 (100)
	Liver	55 (100)
	Abdominal wall	54 (98.2)
	Urogenital	
	Kidneys	53 (96.4)
	Bladder	55 (100)
	Genital tubercle	53 (96.4)
	Other	
	Nuchal membrane	55 (100)
	Skin over spine	53 (96.4)
	Umbilical cord	55 (100)
	Gestational sac	55 (100)
	Yolk sac	22 (40.0)
	Musculoskeletal	
	Cranium	55 (100)
	Frontal bones	55 (100)
	Orbita	55 (100)
	Nasal bone	55 (100)
	Vomer	51 (92.7)
	Maxilla	55 (100)
	Mandible	55 (100)
	Occipital bone	55 (100)
	Clavicle	55 (100)
	Scapula	53 (96.4)
	Spine	55 (100)
	Ribs	55 (100)
	Humerus	55 (100)
	Ulna	55 (100)
	Radius	55 (100)
	Metacarpals	54 (98.2)
	Femur	55 (100)
	Fibula	55 (100)
	Tibia	55 (100)
	Metatarsals	35 (63.6)
	Phalanges feet	29 (52.7)
	Phalanges hands	52 (94.5)
	Diaphragm	55 (100)

A total of 55 pregnancies were used for the 3D VR evaluation.

3D = 3-dimensional; US = ultrasound; VR = virtual reality.

* Not included in our literature search, added to the checklist afterward based on offline VR assessment.

Finally, the checklist consists of a total of 84 different ultrasonically discernible fetal structures. All structures were classified into 11 different subgroups according to the function and/or location of the described structure.

### Offline assessment using 3D US combined with VR

Table 3 provides an overview of the (maternal and fetal) background characteristics of the 55 selected pregnancies at the time of US dataset acquirement. The mean maternal age was 26.4 years (SD 5.2 years) and the mean BMI was 23.8 kg/m^2^ (SD 0.6 kg/m^2^). Seven (12.7%) women conceived after IVF/ICSI.

**Table 3.** Background characteristics selected 3D and 4D STIC ultrasound datasets (n = 55).

	Mean (SD)/n (%)	95% CI for mean
Maternal		
Maternal age (years)	26.4 (SD 5.2)	25.0–28.8
BMI (kg/m^2^)	23.8 (SD 0.6)	22.7–24.9
Ethnicity		
West-European	48 (87.3%)	
Other	7 (12.7%)	
Nulliparous	45 (81.8%)	
Folic acid supplement use	54 (98.2%)	
Smoking, yes (cigarettes)	5 (9.1%)	
Conception		
Natural conception	48 (87.3%)	
IVF/ICSI/OI/IUI	7 (12.7%)	
Reason of prenatal diagnostics		
1st degree family member with CA	36 (65.5%)	
MCDA twins	1 (1.8%)	
Teratogenic drug use	7 (12.7%)	
Maternal disease	6 (10.9%)	
IVF/ICSI	5 (9.1%)	
Fetal*		
Gestational age (weeks ^+ days^)	12^+6^ (SD 0.4 days)	12^+6^–13^+0^
Crown-rump length (mm)	66.0 (SD 0.8 mm)	64.4–67.6
Ultrasound approach		
Transabdominal	3 (5.4%)	
Transvaginal	29 (52.7%)	
Both	23 (41.8%)	
Quality score 3D US datasets		
Average (score 3)	15 (27.3%)	
High (score 4)	40 (72.7%)	
Quality score 4D STIC US datasets		
Low (score 1 or 2)	17 (30.9%)	
Average (score 3)	19 (34.5%)	
High (score 4)	19 (34.5%)	

OI = ovulation induction; CA = congenital anomaly; MCDA = monochorionic diamniotic; 3D = 3-dimensional; US = ultrasound; 4D = 4-dimensional; STIC = spatio-temporal image correlation.

* In our cohort, only datasets from fetuses without congenital anomalies were selected.

The mean GA at US examination was 12^+6 ^weeks (SD 0.4 days) and the mean CRL 66.0 mm (SD 0.8 mm). The US approach was TA, TV, or a combination in respectively 3 (5.4%), 29 (52.7%), and 23 (41.8%) datasets. The quality score of the 3D US datasets was high (score 4) in 40 and average (score 3) in 15 datasets. The quality score of the 4D STIC datasets was 1 in 14.5% (n = 8), 2 in 16.4% (n = 9), 3 in 34.5% (n = 19), and 4 in 34.5% (n = 19).

**Figure 3.**
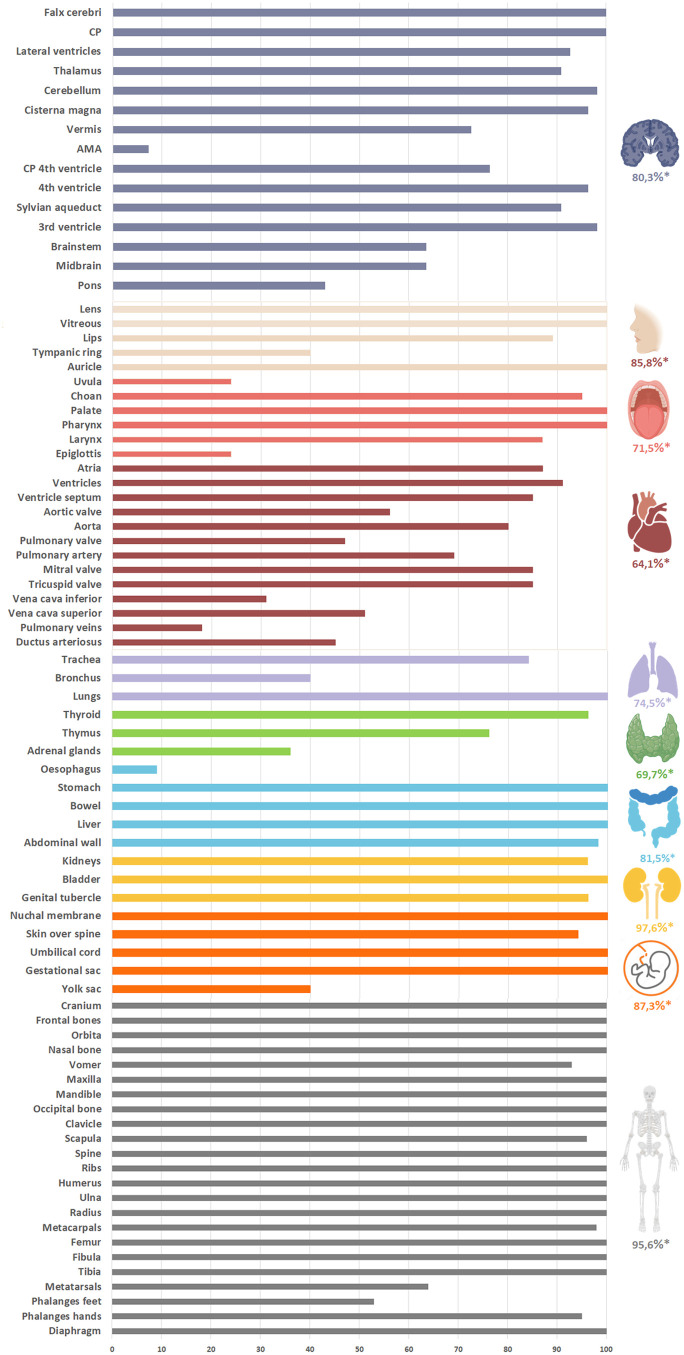
**Visibility rate per structure and mean visibility rate per subgroup using 3-dimensional ultrasound combined with virtual reality.** A total of 55 pregnancies were used for the evaluation of each organ system according to the constructed checklist (overall mean visibility rate 82.2%). *Mean visibility rate (%), CP (Choroid plexus), AMA (Anterior membranous area).

After performance of the offline assessment using 3D US combined with VR, the visibility rate (representing the percentage as a proportion of the total number of assessed US datasets (n = 55)) per structure was calculated. Figure 3 displays the visibility rate per structure and the mean visibility rate per subgroup (based on the function and/or location of the described structure). Column 2 of Table 2 represents the corresponding visibility rates displayed in numbers and percentages per structure. The mean visibility rate for all 84 structures incorporated in the checklist was 82.2%. From all subgroups, anatomical structures representing the urogenital system reached the highest mean visibility rate (97.6%). The lowest mean visibility rate was reached for the cardiovascular system (64.1%). Structures with a visibility rate of 100% (visualized in all 55 selected pregnancies) were: the choroid plexus, falx cerebri, liver, stomach, bowel, palate, pharynx, lungs, bladder, the majority of the skeleton, eyes (vitreous body and lens), ears, and extra-fetal structures such as the gestational sac and the umbilical cord. Following offline VR assessment, three structures not initially included in our literature search were identified in 40.0% (main bronchus in 22/55), 23.6% (uvula in 13/55), and 23.6% (epiglottis in 13/55) of all evaluated 3D US datasets. Examples of fetal structures reported in literature that were less frequently observed (mean visibility rate <30%) during 3D VR assessment were the pulmonary veins and esophagus.

## Discussion

### Principal findings

A comprehensive overview of first-trimester physiological fetal anatomy discernable by US was developed based on a systematic literature review. Subsequently, a prospective clinical evaluation was conducted to demonstrate the applicability of 3D US combined with VR for the offline assessment of fetal anatomy during the first trimester of pregnancy.

All fetal structures included in the checklist were discernible using 3D US combined with VR, with a mean visibility rate of 82.2%.

### Strengths and limitations

Reflecting on our findings, we believe that the mean visibility rate of 82% is primarily limited by the prioritization of 3D US datasets quality over 4D STIC datasets quality, potentially leading to the use of lower-quality 4D STIC datasets for assessing cardiovascular structures from the same fetus. In addition, it must be taken into account that the 3D and 4D US datasets were obtained during a complete fetal US evaluation without the performance of targeted US examination. We believe that the implementation of targeted scanning will even further enhance the quality of both 3D and 4D US datasets, resulting in an expected increase in the actual visibility rate compared to what is currently observed.

Besides optimal visualization through real depth perception as the most significant advantage of 3D US combined with VR, the possibility of offline analysis offers an additional major benefit. Although it is possible to review 2D data for the assessment of anatomical structures after storage, the added value of stored 2D datasets for offline analysis is limited. A stored 2D dataset essentially represents a static image, meaning that offline analysis can only be conducted on fetal anatomical structures included in the image, further constrained by the acquisition plane prior to storage. Offline analysis of 3D data using VR, in contrast, enables limitless interaction, magnification, and visualization of any eligible plane throughout the fetus. In theory, 3D VR offers the same advantages as live scanning, but without the associated limitations.

Ideally, offline analysis could eliminate the need for a comprehensive assessment of the fetus during the initial visit, as the ability to analyze 3D or 4D datasets post-acquisition may provide greater flexibility and accuracy. This also raises questions regarding the demand placed on obstetric health care providers to make accurate diagnoses ‘on the spot’ (directly following a fetal US examination), considering that diagnostic imaging in other medical fields typically involves an offline analysis where a diagnosis is made afterward.

Furthermore, as a result of the storage of 3D and 4D US datasets, VR-based visualization can be deployed remotely at any location and time, such as for expert consultation.

3D and 4D US datasets were prospectively collected and selected based on quality. Which can be seen as a limitation as quality selection of US datasets has the potential to introduce selection bias, possibly leading to an overestimation of the detection rate of 3D US combined with VR. Additionally, the extrapolation of structure identification to the general population may be impeded as our study population was recruited from a tertiary referral center and US examinations were performed by experienced sonographers using a high‐frequency TV US transducer. However, such a setting is inevitable as to acquire accurate understanding of fetal anatomy for enhanced detection of first-trimester fetal (patho-)physiology using 3D US combined with VR.

### Explanation of results and implications for clinical practice

Based on the available literature we can conclude that US in the first trimester of pregnancy is already extensively applied as a non-invasive *in vivo* tool for fetal anatomical assessment in routine obstetric care. Nevertheless, from all studies included (372 out of 15 874 yielded studies) for the construction of our checklist, only seven studies used 3D US combined with VR (1.9%). All these studies were performed at the Erasmus Medical Center, using participants from one ongoing hospital-based cohort (The Rotterdam Periconceptional Cohort (Predict Study)) and one population-based cohort (Generation R Next Study). The limited number of studies using 3D US combined with VR can be attributed to the challenges associated with its implementation in clinical practice.

However, to facilitate the implementation of 3D VR in an outpatient clinical setting, a VR desktop system—using the same V-Scope software as used for the I-Space—was developed and validated (Baken *et al.*, 2015). The VR desktop system provides the same tools and functions as described for the I-Space but is less expensive and has fewer logistical constraints, using a regular personal computer, a 3D monitor, and an optical tracking system.

### Future implications

Regarding future perspectives, 3D VR could be used as a new modality in hospitals as an adjunct tool to specialist care (targeted cases) for conducting diagnostics toward fetal anomalies and serving as an educating and counseling tool for parents expecting a child with a fetal anomaly. Additionally, VR can facilitate offline or remote analysis and provide expert advice for referral centers when there is diagnostic uncertainty. In both scenarios, VR could also serve as a valuable tool for training purposes.

In the future, 3D VR could be integrated into screening settings to assist in the (semi-)automatic identification of potential aberrant growth trajectories and structural anomalies. Additionally, we believe that the prospect of (semi-) automated innovative volumetric measurements (i.e. fetal proportional- or brain measurements; Koning *et al.*, 2009; Rousian *et al.*, 2011; Baken *et al.*, 2014; Muhanna, 2015; Pietersma *et al.*, 2020; Wiertsema *et al.*, 2021) facilitates the development of novel deep learning methods of US techniques with ultimately, the aim to develop an ‘artificially intelligent’ instrument for fetal anomaly screening in the first trimester of pregnancy.(Bastiaansen *et al.*, 2020) Finally, considering its societal significance, 3D VR could offer additional value by integrating emerging AI models to help address the medical labor shortage.

## Conclusion

This is the first comprehensive literature-based overview of all ultrasonically discernible fetal anatomy in the first trimester of pregnancy which has been evaluated in a prospective clinical setting.

Moreover, this study underlines the applicability of 3D US combined with VR, both as an instrument to assess fetal anatomy in the first trimester and a reference resource for professionals.

## Supplementary Material

deaf112_Supplementary_Data_File_S1

deaf112_Supplementary_Data_File_S2

deaf112_Supplementary_Figure_S1

deaf112_Supplementary_Table_S1

deaf112_Supplementary_Table_S2

deaf112_Supplementary_Video_S1

## Data Availability

The data that support the findings of this study are available from the corresponding author upon reasonable request.
