# Supplementary material for: An overview and evaluation of first-trimester physiological fetal human anatomy using 3-dimensional ultrasound combined with virtual reality techniques
Source: Hum Reprod. 2025 Jun 27;40(8):1495–503. doi: 10.1093/humrep/deaf112 (PMC12378615; doi:10.1093/humrep/deaf112)
Supplement: deaf112_Supplementary_Data_File_S2 [file deaf112_Supplementary_Data_File_S2.pdf]

OXFORD
human reproduction

Human Reproduction, 2025, 1–1
https://doi.org/10.1093/humrep/deaf112
Supplementary Data

Supplementary Data File S2

The Newcastle-Ottawa Quality assessment adapted for cross-sectional studies.

NEWCASTLE—OTTAWA QUALITY ASSESSMENT SCALE CROSS-SECTIONAL STUDIES

Selection

1. Representativeness of the sample
   a) Truly representative of the average in the target population* (all subjects or random sampling)
   b) Somewhat representative of the average in the target group* (non-random sampling)
   c) Selected group of users/convenience sample
   d) No description of the derivation of the included subjects
2. Sample size
   a) Justified and satisfactory (including sample size calculation)*
   b) Not justified
   c) No information provided
3. Non-respondents
   a) Proportion of target sample recruited attains pre-specified target or basic summary of non-respondent characteristics in sampling frame recorded*
   b) Unsatisfactory recruitment rate, no summary data on non-respondents
   c) No information provided
4. Ascertainment of the exposure (risk factor)
   a) Vaccine records/vaccine registry/clinic registers/hospital records only. **
   b) Parental or personal recall and vaccine/hospital records. *
   c) Parental/personal recall only.

Comparability (Maximum 2 stars)

1. Comparability of subjects in different outcome groups on the basis of design or analysis. Confounding factors controlled.
   a) Data/results adjusted for relevant predictors/risk factors/confounders, e.g. age, sex, time since vaccination, etc.**
   b) Data/results not adjusted for all relevant confounders/risk factors/information not provided.

Outcome

1. Assessment of outcome
   a) Independent blind assessment using objective validated laboratory methods**
   b) Unblinded assessment using objective validated laboratory methods**
   c) Used non-standard or non-validated laboratory methods with gold standard*
   d) No description/non-standard laboratory methods used.
2. Statistical test:
   a) Statistical test used to analyze the data clearly described, appropriate and measures of association presented including confidence intervals and probability level (*P*-value)*
   b) Statistical test not appropriate, not described or incomplete.

This scale has been adapted from the Newcastle-Ottawa Quality Assessment Scale for cohort studies to provide quality assessment of cross sectional studies[1].

1. Herzog R, *et al.* Is healthcare workers' intention to vaccinate related to their knowledge, beliefs and attitudes? A systematic review. *BMC Public Health* 2013;**13**:154.
