# Supplementary material for: An overview and evaluation of first-trimester physiological fetal human anatomy using 3-dimensional ultrasound combined with virtual reality techniques
Source: Hum Reprod. 2025 Jun 27;40(8):1495–503. doi: 10.1093/humrep/deaf112 (PMC12378615; doi:10.1093/humrep/deaf112)
Supplement: deaf112_Supplementary_Figure_S1 [file deaf112_Supplementary_Figure_S1.pdf]

OXFORD
human reproduction

Human Reproduction, 2025, 1–1
https://doi.org/10.1093/humrep/deaf112
Supplementary Data

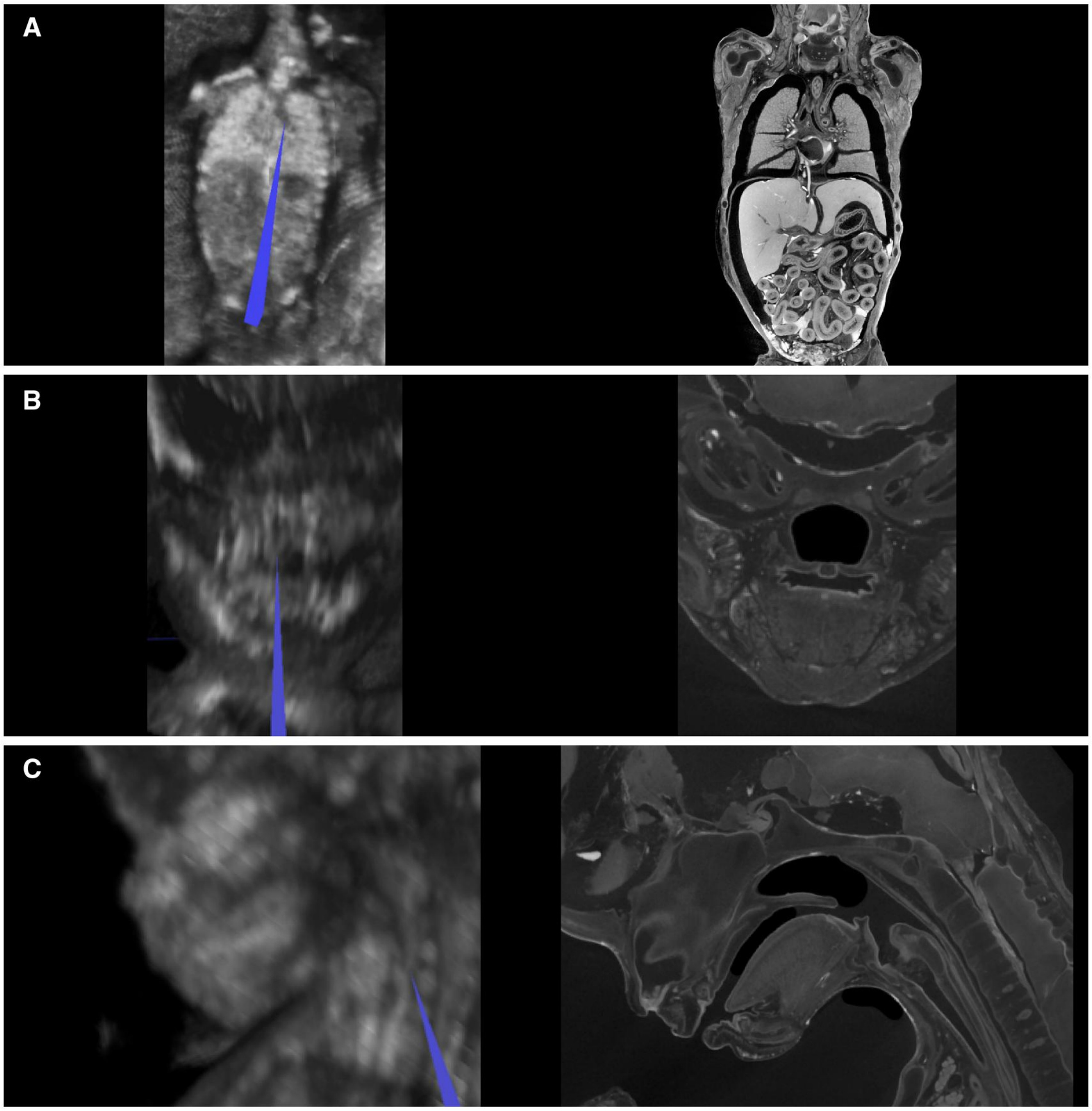

Supplementary Figure S1. Fetal anatomical structures, not included in our literature search, displayed in left: 3-dimensional ultrasound and virtual reality, right: micro-CT scans of histologically sectioned human fetus from the Dutch fetal biobank. (**A**) A coronal section of the fetal thorax and abdomen demonstrating the presence of the left main bronchus, indicated by the blue arrow. (**B**) A coronal section of the fetal head demonstrating the presence of the uvula, indicated by the blue arrow. (**C**) A sagittal section of the fetal head and neck demonstrating the presence of epiglottis, indicated by the blue arrow.
