# Supplementary material for: An overview and evaluation of first-trimester physiological fetal human anatomy using 3-dimensional ultrasound combined with virtual reality techniques
Source: Hum Reprod. 2025 Jun 27;40(8):1495–503. doi: 10.1093/humrep/deaf112 (PMC12378615; doi:10.1093/humrep/deaf112)
Supplement: deaf112_Supplementary_Table_S1 [file deaf112_Supplementary_Table_S1.pdf]

OXFORD human reproduction

*Human Reproduction*, 2025, 1–2
https://doi.org/10.1093/humrep/deaf112
Supplementary Data

**Supplementary Table S1.** Literature search string (conducted 12 January 2024).

| |
|---|
| **Embase** |
| ('first trimester pregnancy'/exp OR (((first OR 1st) NEAR/3 trimester*) OR (((11 OR 12 OR 13 OR 11th OR 12th OR 13th OR eleven* OR twelve* OR twelfth* OR thirteen*) NEXT/1 (week*)) AND (pregnan* OR gestation* OR prenatal* OR antenatal* OR prepart*)) OR (early NEAR/3 pregnan*)):ab, ti, kw) AND ('echography'/de OR 'Doppler echography'/de OR echocardiography/exp OR 'echograph'/de OR 'fetus echography'/de OR 'transvaginal echography'/exp OR 'ultrasound'/de OR 'color ultrasound flowmetry'/de OR 'Doppler flowmetry'/de OR 'three dimensional imaging'/exp OR 'two-dimensional imaging'/de OR 'four-dimensional imaging'/de OR (echogra* OR echocardiograph* OR ultraso* OR ultra-so* OR sonogra* OR Vaginosonogra* OR doppler* OR scan OR ((three OR 3 OR two OR 2 OR four OR 4) NEXT/1 dimension*) OR 3d-imag* OR 2d-imag* OR 4d-imag*):ab, ti, kw) AND ('anatomy'/exp OR 'anatomical variation'/de OR 'head'/de OR 'congenital disorder'/de OR 'congenital malformation'/exp OR 'feature detection'/de OR 'brain'/exp OR 'kidney'/exp OR 'umbilical cord'/exp OR 'spine'/exp OR 'neck'/exp OR 'heart'/exp OR 'lung'/exp OR 'diaphragm'/exp OR 'stomach'/exp OR 'intestine'/exp OR 'gallbladder'/exp OR 'bladder'/exp OR 'limb'/exp OR 'genital system'/exp OR 'digestive system'/exp OR 'body regions'/exp OR 'anatomic landmark'/de OR 'musculoskeletal system'/exp OR 'deformity'/exp OR 'fetus development'/exp OR (anatom* OR abnormal* OR structure OR structures OR structural OR anomal* OR head OR brain OR cerebr* OR kidney* OR umbilical* OR spine OR spinal OR neck* OR heart OR cardiac* OR lung* OR pulmo* OR diaphragm* OR stomach* OR intestin* OR bowel* OR duoden* OR gallbladder* OR bladder* OR limb* OR extremit* OR genital* OR esophag* OR eosophag* OR finger* OR toe OR toes OR digit* OR birth-defect* OR malformat* OR abdomen OR abdominal OR pelvic* OR thoracic OR thorax OR organ OR organs OR pelvis OR bone* OR muscle* OR digestiv* OR alimentar* OR gastrointestin* OR pancrea* OR aort* OR vessel* OR arter* OR umbilic* OR trachea* OR respirator* OR deformit* OR deformation OR disfigure* OR ((congenital) NEAR/3 (disorder* OR defect*)) OR ((feature*) NEAR/3 (detect* OR doppler* OR ultraso* OR echo* OR sonog* OR scan*)) OR ((fetus OR fetal OR foetus OR foetal) NEAR/3 (developm* OR growth OR matur*))):ab, ti, kw) NOT ((animal/exp OR animal*:de OR nonhuman/de) NOT ('human'/exp)) AND ([dutch]/lim OR [english]/lim) NOT ([Conference Abstract]/lim) |
| **Medline** |
| (Pregnancy Trimester, First/OR (((first OR 1st) ADJ3 trimester*) OR (((11 OR 12 OR 13 OR 11th OR 12th OR 13th OR eleven* OR twelve* OR twelfth* OR thirteen*) ADJ (week*)) AND (pregnan* OR gestation* OR prenatal* OR antenatal* OR prepart*)) OR (early ADJ3 pregnan*)).ab, ti, kf.) AND (Ultrasonography/OR exp Ultrasonography, Doppler/OR exp Echocardiography/OR exp Ultrasonography, Prenatal/OR Ultrasonics/ OR Imaging, Three-Dimensional/OR (echogra* OR echocardiograph* OR ultraso* OR ultra-so* OR sonogra* OR Vaginosonogra* OR doppler* OR scan OR ((three OR 3 OR two OR 2 OR four OR 4) ADJ dimension*) OR 3d-imag* OR 2d-imag* OR 4d-imag*).ab, ti, kf.) AND (Anatomy/OR Anatomy, Cross-Sectional/OR Anatomy, Regional/OR Osteology/OR exp head/OR exp "Congenital, Hereditary, and Neonatal Diseases and Abnormalities"/OR epx Brain/OR exp Kidney/OR exp Torso/OR exp Neck/OR exp Cardiovascular System/OR exp Respiratory System/OR exp Digestive System/OR exp Urogenital System/OR Body Regions/OR exp Extremities/OR Anatomic Landmarks/OR exp Musculoskeletal System/ OR exp Fetal Development/OR (anatom* OR abnormal* OR structure OR structures OR structural OR anomal* OR head OR brain OR cerebr* OR kidney* OR umbilical* OR spine OR spinal OR neck* OR heart OR cardiac* OR lung* OR pulmo* OR diaphragm* OR stomach* OR intestin* OR bowel* OR duoden* OR gallbladder* OR bladder* OR limb* OR extremit* OR genital* OR esophag* OR eosophag* OR finger* OR toe OR toes OR digit* OR birth-defect* OR malformat* OR abdomen OR abdominal OR pelvic* OR thoracic OR thorax OR organ OR organs OR pelvis OR bone* OR muscle* OR digestiv* OR alimentar* OR gastrointestin* OR pancrea* OR aort* OR vessel* OR arter* OR umbilic* OR trachea* OR respirator* OR deformit* OR deformation OR disfigure* OR ((congenital) ADJ3 (disorder* OR defect*)) OR ((feature*) ADJ3 (detect* OR doppler* OR ultraso* OR echo* OR sonog* OR scan*)) OR ((fetus OR fetal OR foetus OR foetal) ADJ3 (developm* OR growth OR matur*))).ab, ti, kf.) NOT (exp animals/NOT humans/) AND (dutch.la. OR english.la.) NOT (news OR congres* OR abstract* OR book* OR chapter* OR dissertation abstract*).pt. |
| **Cochrane** |
| ((((first OR 1st) NEAR/3 trimester*) OR (((11 OR 12 OR 13 OR 11th OR 12th OR 13th OR eleven* OR twelve* OR twelfth* OR thirteen*) NEXT/1 (week*)) AND (pregnan* OR gestation* OR prenatal* OR antenatal* OR prepart*)) OR (early NEAR/3 pregnan*)):ab, ti) AND ((echogra* OR echocardiograph* OR ultraso* OR ultra NEXT so* OR sonogra* OR Vaginosonogra* OR doppler* OR scan OR ((three OR 3 OR two OR 2 OR four OR 4) NEXT/1 dimension*) OR 3d NEXT imag* OR 2d NEXT imag* OR 4d NEXT imag*):ab, ti) AND ((anatom* OR abnormal* OR structure OR structures OR structural OR anomal* OR head OR brain OR cerebr* OR kidney* OR umbilical* OR spine OR spinal OR neck* OR heart OR cardiac* OR lung* OR pulmo* OR diaphragm* OR stomach* OR intestin* OR bowel* OR duoden* OR gallbladder* OR bladder* OR limb* OR extremit* OR genital* OR esophag* OR eosophag* OR finger* OR toe OR toes OR digit* OR birth NEXT defect* OR malformat* OR abdomen OR abdominal OR pelvic* OR thoracic OR thorax OR organ OR organs OR pelvis OR bone* OR muscle* OR digestiv* OR alimentar* OR gastrointestin* OR pancrea* OR aort* OR vessel* OR arter* OR umbilic* OR trachea* OR respirator* OR deformit* OR deformation OR disfigure* OR ((congenital) NEAR/3 (disorder* OR defect*)) OR ((feature*) NEAR/3 (detect* OR doppler* OR ultraso* OR echo* OR sonog* OR scan*)) OR ((fetus OR fetal OR foetus OR foetal) NEAR/3 (developm* OR growth OR matur*))):ab, ti) NOT ("conference abstract":kw OR Trial registry record: pt) |
| **Web of Science 6395** |
| TS=(((((first OR 1st) NEAR/2 trimester*) OR (((11 OR 12 OR 13 OR 11th OR 12th OR 13th OR eleven* OR twelve* OR twelfth* OR thirteen*) NEAR/1 (week*)) AND (pregnan* OR gestation* OR prenatal* OR antenatal* OR prepart*)) OR (early NEAR/2 pregnan*))) AND ((echogra* OR echocardiograph* OR ultraso* OR ultra-so* OR sonogra* OR Vaginosonogra* OR doppler* OR scan OR ((three OR 3 OR two OR 2 OR four OR 4) NEAR/1 dimension*) OR 3d-imag* OR 2d-imag* OR 4d-imag*)) AND ((anatom* OR abnormal* OR structure OR structures OR structural OR anomal* OR head OR brain OR cerebr* OR kidney* OR umbilical* OR spine OR spinal OR neck* OR heart OR cardiac* OR lung* OR pulmo* OR diaphragm* OR stomach* OR intestin* OR bowel* OR duoden* OR gallbladder* OR bladder* OR limb* OR extremit* OR genital* OR esophag* OR eosophag* OR finger* OR toe OR toes OR digit* OR birth-defect* OR malformat* OR abdomen OR abdominal OR pelvic* OR thoracic OR thorax OR organ OR organs OR pelvis OR bone* OR muscle* OR digestiv* OR alimentar* OR gastrointestin* OR pancrea* OR aort* OR vessel* OR arter* OR umbilic* OR trachea* OR respirator* OR deformit* OR deformation OR disfigure* OR ((congenital) NEAR/2 (disorder* OR defect*)) |

(continued)

**Supplementary Table S1.** Continued

| Web of Science 6395 |
| --- |
| OR ((feature*) NEAR/2 (detect* OR doppler* OR ultraso* OR echo* OR sonog* OR scan*)) OR ((fetus OR fetal OR foetus OR foetal) NEAR/2 (developm* OR growth OR matur*)))) NOT ((animal* OR rat OR rats OR mouse OR mice OR murine OR dog OR dogs OR canine OR cat OR cats OR feline OR rabbit OR cow OR cows OR bovine OR rodent* OR sheep OR ovine OR pig OR swine OR porcine OR veterinar* OR chick* OR zebrafish* OR baboon* OR nonhuman* OR primate* OR cattle* OR goose OR geese OR duck OR macaque* OR avian* OR bird* OR fish*) NOT (human* OR patient* OR women OR woman OR men OR man))) NOT DT=(Meeting Abstract OR Meeting Summary) AND LA=(English OR Dutch) |
| **Google Scholar (200 top ranked)** |
| “first\|1st trimester” echography\|echocardiography\|ultrasound\|sonography\|doppler\|scan anatomy\|anatomical\|abnormal\|structure\|structures\|structural\|feature\|anomaly\|defect\|malformation\|deformity\|deformation\|“congenital disorder\|defect”<br>‘first\|1st trimester’ echography\|echocardiography\|ultrasound\|sonography\|doppler\|scan anatomy\|anatomical\|abnormal\|structure\|structures\|structural\|feature\|anomaly\|defect\|malformation\|deformity\|deformation\|‘congenital disorder\|defect’ |

© The Author(s) 2025. Published by Oxford University Press on behalf of European Society of Human Reproduction and Embryology.
This is an Open Access article distributed under the terms of the Creative Commons Attribution License (https://creativecommons.org/licenses/by/4.0/), which permits unrestricted reuse, distribution, and reproduction in any medium, provided the original work is properly cited.
Human Reproduction, 2025, 00, 1–15
https://doi.org/10.1093/humrep/deaf112
Original Article
