# Supplementary material for: An overview and evaluation of first-trimester physiological fetal human anatomy using 3-dimensional ultrasound combined with virtual reality techniques
Source: Hum Reprod. 2025 Jun 27;40(8):1495–503. doi: 10.1093/humrep/deaf112 (PMC12378615; doi:10.1093/humrep/deaf112)
Supplement: deaf112_Supplementary_Table_S2 [file deaf112_Supplementary_Table_S2.docx]

**Supplementary Table S2.** An overview of included studies, classified per subgroup according to the function and/or location of the described structure, including the name of the first author, the year of publication and gestational age.

| **Subgroup** | **First author**^(1-250)(251-372)^* | **Year** | **GA^†^ in weeks^+days^ (range / mean)** |
| --- | --- | --- | --- |
| Central nervous system | Kushnir | [1989](https://doi.org/10.1002/uog.8965) | 12-14 |
|  | Blaas | [1994](https://doi.org/10.7863/jum.1994.13.10.783) | 7^+6^-11^+4^ |
|  | Blaas | [1995](https://doi.org/10.1002/uog.9068) | 7-12 |
|  | Blaas | 1995 | 7-10 |
|  | Van Zalen-Sprock | [1996](https://doi.org/10.7863/jum.2011.30.5.695) | 7-14 |
|  | Guariglia | [1998](https://doi.org/10.1002/uog.9068) | 11-16 |
|  | Rosati | [1999](https://doi.org/10.1002/pd.1442) | 11-16 |
|  | Singhakom | [2004](https://doi.org/10.7863/ultra.34.1.143) | >13 |
|  | Mittal | [2007](https://doi.org/10.1080/14767058.2019.1637849) | 12-41 |
|  | Tonni | 2008 | 6^+4^ - 12 |
|  | Mi | [2008](https://doi.org/10.1016/j.ajog.2004.03.007) | 7^+4^-13 |
|  | Chaoui | [2009](https://doi.org/10.1002/pd.1442) | 11-13^+6^ |
|  | Pistorius | [2009](https://doi.org/10.1002/uog.51) | 8-10^+6^ |
|  | Hata | [2009](https://doi.org/10.1080/14767058.2020.1718643) | 7-9 |
|  | Xiong | [2010](https://doi.org/10.1002/uog.3948) | 11-13^+6^ |
|  | Egle | 2011 | 11^+2^-14^+1^ |
|  | Finn | [2011](https://doi.org/10.1002/uog.6374) | 11-13^+6^ |
|  | Solt | [2011](https://doi.org/10.1002/uog.5262) | 11-13^+6^ |
|  | Scheier | [2011](https://doi.org/10.7863/ultra.34.1.143) | 11^+4^-13^+4^ |
|  | Chaoui | [2011](https://doi.org/10.7863/ultra.34.1.143) | 11-13 |
|  | Lachmann | 2011 | 11-13+6 |
|  | Mangione | [2011](https://doi.org/10.1002/uog.3948) | 11-13 |
|  | Beamon | [2012](https://dx.doi.org/10.1136%2Fhrt.2003.015065) | 11-13^+6^ |
|  | Kavalakis | [2012](https://doi.org/10.1515/jpm-2019-0222) | 11-14 |
|  | Chen | [2012](https://doi.org/10.1002/uog.9068) | 11^+5^-13^+4^ |
|  | Lee | [2012](https://doi.org/10.1016/j.ajog.2004.03.007) | 10^+5^-12^+6^ |
|  | Ergin | [2012](https://doi.org/10.1080/14767058.2020.1718643) | 11^+0^-13^+6^ |
|  | Loureiro | 2012 | 11-13 |
|  | Loureiro | 2012 | 11-13 |
|  | Rousian | [2013](https://doi.org/10.1046/j.1469-0705.1999.14050307.x) | 6-12 |
|  | Garcia-Posada | 2013 | 11-13^+6^ |
|  | Abu-Rustrum | [2013](https://doi.org/10.1002/uog.3948) | 11^+3^-14^+1^ |
|  | Fong | [2014](https://doi.org/10.1080/14767058.2019.1637849) | 11-13 |
|  | Yuksel | [2014](https://doi.org/10.7863/jum.1994.13.10.783) | 11-14 |
|  | Gijtenbeek | [2014](https://doi.org/10.1016/j.echo.2017.03.017) | 6-12^+6^ |
|  | Hsiao | [2015](https://doi.org/10.7863/ultra.34.1.143) | 11-13 |
|  | Boitor-Borza | [2015](https://doi.org/10.7863/jum.2011.30.5.695) | 9-13 |
|  | Kappou | [2015](https://doi.org/10.1002/pd.1442) | 11-13 |
|  | Gindes | [2015](https://doi.org/10.7863/ultra.34.1.143) | 12-33 |
|  | Liu | [2015](https://doi.org/10.1002/uog.9068) | 11-13^+6^ |
|  | Peixoto | [2016](https://doi.org/10.1080/14767058.2020.1718643) | 11-14^+2^ |
|  | Manegold-Brauer | 2016 | 11-14 |
|  | Altmann | 2016 | 11-13^+6^ |
|  | Molina-Giraldo | [2016](https://doi.org/10.7863/jum.1994.13.10.783) | 11^+0^-13^+6^ |
|  | Nanthakomon | [2016](https://doi.org/10.1002/pd.1442) | 11^+0^-13^+6^ |
|  | Ponmozhi | 2017 | 11-14 |
|  | Shah | [2017](https://doi.org/10.1016/0301-5629(93)90120-D) | 13^+1^ |
|  | Koning | [2017](https://doi.org/10.1002/uog.3948) | 9-32 |
|  | Teegala | 2017 | 11-13^+6^ |
|  | Yang | 2017 | 10^+6^-13^+6^ |
|  | Parisi | [2018](https://doi.org/10.7863/jum.2012.31.4.563) | 9-32 |
|  | Altmann | [2018](https://doi.org/10.1002/pd.1442) | 11^+2^-14^+1^ |
|  | Kose | [2018](https://doi.org/10.1002/pd.1442) | 11-13 |
|  | Ferreira | [2019](https://doi.org/10.7863/jum.2012.31.4.563) | 11-13 |
|  | Comanescu | [2019](https://doi.org/10.7863/ultra.34.1.143) | 11^+6^-13^+4^ |
|  | Pauta | 2019 | 11-14 |
|  | Garcia-Rodriguez | [2019](https://doi.org/10.1067/mob.2003.157) | 11-13 |
|  | Chaoui | [2020](https://doi.org/10.7863/ultra.34.1.143) | 11-13^+6^ |
|  | Eriç Özdemir | [2021](https://doi.org/10.1016/j.ajog.2004.03.007) | 11-14 |
|  | Ramirez Zegarra | [2021](https://doi.org/10.7863/ultra.34.1.143) | 11-13^+6^ |
|  | Volpe | [2021](https://doi.org/10.1080/14767058.2020.1718643) | 12-13 |
|  | Altmann | [2022](https://doi.org/10.7863/jum.2012.31.4.563) | 11^+3^-13^+6^ |
|  | Husen | [2022](https://doi.org/10.1002/pd.1442) | 9-11 |
|  | Altmann | 2023 | 11^+3^-13^+6^ |
|  | Liu | 2023 | 7-13^+6^ |
|  | Manzo | 2023 | 13-39 |
|  | Weissbach | 2023 | 13-19 |
| Face | Christ | 1983 | 11-38 |
|  | Achiron | 1995 | 12-17 |
|  | Sacchini | [2003](http://dx.doi.org/10.1136/hrt.77.1.68) | 11-14 |
|  | Placensia | 2007 | 11-13^+6^ |
|  | Placensia | 2007 | 11-13^+6^ |
|  | Borenstein | [2007](https://doi.org/10.1159/000020970) | 11-13^+6^ |
|  | McGahan | 2008 | 11-38 |
|  | Alphonse | 2010 | 11-13^+6^ |
|  | Jeon | 2010 | 11-13^+6^ |
|  | Vicario | 2010 | 11-13^+6^ |
|  | Yang | 2010 | 11-13 |
|  | Sepulveda | 2010 | 11-13 |
|  | Ginsberg | 2011 | 11-13^+6^ |
|  | Chen | 2011 | 11-13^+6^ |
|  | Leibovitz | 2013 | 11^+1^-12^+6^ |
|  | Panigassi | 2013 | 11-13^+6^ |
|  | Hsiao | 2013 | 11-13^+6^ |
|  | Alphonse | 2013 | 11-13^+6^ |
|  | Zajicek | 2013 | 12-16 |
|  | Shyu | 2014 | 11-13^+6^ |
|  | Alphonse | 2014 | 11^+3^-14 |
|  | Pranpanus | 2016 | 11-13^+6^ |
|  | Ji | 2021 | 11-13^+6^ |
|  | Ji | 2022 | 11-11^+6^ |
|  | Li | 2022 | 11-13^+6^ |
|  | Zhou | 2022 | 11-13^+6^ |
| Oropharangeal | Bronshtein | 1998 | 13-18 |
|  | Sepulveda | 2012 | 11-13 |
|  | Liberty | [2013](https://doi.org/10.7863/jum.2011.30.5.695) | 11-13^+6^ |
|  | Marginean | 2013 | 11-13^+6^ |
|  | Yazdi | [2013](https://doi.org/10.7863/ultra.34.1.143) | 11-13^+6^ |
|  | Hoopmann | [2016](https://doi.org/10.1002/pd.1442) | 11-13^+6^ |
|  | Lakshmy | [2017](https://doi.org/10.1046/j.1469-0705.2002.00735.x) | 11^+6^-14 |
|  | Wojtowicz | [2018](https://doi.org/10.1515/jpm-2019-0222) | 11-13^+6^ |
| Cardiovascular | D'Amelio | 1991 | 11-14 |
|  | Johnson | 1992 | 10-16 |
|  | Brezinka | 1993 | 9-25 |
|  | Achiron | 1994 | 13-15 |
|  | Allan | 1997 | 5-12 |
|  | Carvalho | 1998 | 12-13^+6^ |
|  | Lam | 1999 | 12-14 |
|  | Leiva | 1999 | 6-14 |
|  | Gembruch | 2000 | 10-17 |
|  | Haak | 2002 | 11-13^+6^ |
|  | Carvalho | 2004 | 10^+1^-13^+6^ |
|  | Huggon | 2004 | 11-14 |
|  | Vimpelli | 2006 | 11-13^+6^ |
|  | Smrcek | 2006 | 10-15 |
|  | Marques Carvalho | 2008 | 11-14^+6^ |
|  | Molina | 2008 | 12-34 |
|  | Viñals | 2008 | 11-13^+6^ |
|  | Uittenbogaard | 2009 | 12-30 |
|  | Bennasar | 2009 | 11-15 |
|  | Turan | 2009 | 11^+1^-13^+6^ |
|  | Sinkovskaya | 2010 | 11-14^+6^ |
|  | Rozmus-Warcholinska | 2010 | 11-14 |
|  | Jadaon | 2011 | 11-34 |
|  | Brestak | 2011 | 11-13^+6^ |
|  | Yuan | 2011 | 11-37 |
|  | Krapp | 2011 | 11^+2^-14^+1^ |
|  | Abu-Rustum | 2011 | 11^+6^-13^+6^ |
|  | Gindes | 2012 | 10^+2^-14^+0^ |
|  | Votino | 2012 | 11-13^+6^ |
|  | Rembouskos | 2012 | 11^+2^-14^+1^ |
|  | McBrien | 2013 | 8-14^+6^ |
|  | Lima | 2013 | 12-14 |
|  | Votino | 2013 | 11-14 |
|  | Pares | 2013 | 12-14 |
|  | Tudorache | 2013 | 12-13^+6^ |
|  | Brestak | 2014 | 11-13^+6^ |
|  | Persico | 2014 | 11^+6-^14^+1^ |
|  | Orlandi | 2014 | 11-14 |
|  | Zhen | 2015 | 11-13^+6^ |
|  | Sinkovskaya | 2015 | 11-14^+6^ |
|  | Nemescu | 2015 | 11^+1^-14^+1^ |
|  | Adekola | 2016 | 11-15 |
|  | Krishnan | 2016 | 12-39 |
|  | Dmitrovic | 2016 | 12-14 |
|  | Yu | 2016 | 11-13^+6^ |
|  | Wanapirak | 2017 | 10-24 |
|  | Hutchinson | 2017 | 6-13 |
|  | Lakshmy | 2017 | 11-14 |
|  | De Robertis | 2017 | 11^+2^-14^+1^ |
|  | Quarello | 2017 | 11^+2^-14^+1^ |
|  | Kayaalti | 2018 | 11^+6^-15^+2^ |
|  | García Fernández | 2019 | 11-13^+6^ |
|  | Majeed | 2019 | 13-15^+6^ |
|  | Ebrashy | 2019 | 11-13^+6^ |
|  | Asoglu | 2019 | 11-14^+6^ |
|  | Zheng | 2019 | 11^+2^-14^+2^ |
|  | Jung | 2020 | 11-13^+6^ |
|  | Kamel | 2020 | 10-13 |
|  | Garcia Delgado | 2021 | 13^+0^-13^+6^ |
|  | Sternfeld | 2021 | 12-39 |
|  | Ye | 2021 | 13^+0^-14^+6^ |
|  | Hata | 2022 | 12-14^+6^ |
|  | Lin | 2022 | 11-13^+6^ |
|  | Patabendige | 2023 | 11-30 |
|  | Tu | 2023 | 11-14 |
| Respiratory | Peralta | 2005 | 12-13^+6^ |
|  | Peralta | [2006](http://dx.doi.org/10.1136/hrt.77.1.68) | 12-13^+6^ |
|  | Lian | [2021](https://doi.org/10.1515/jpm-2019-0222) | 13-40 |
| Endocrine glands | Bronshtein | 1993 | 12-17 |
|  | Radaelli | 2002 | 12-14 |
|  | Weissmann-Brenner | 2015 | 13-16 |
|  | Borgelt | 2016 | 11-13^+6^ |
|  | Basaran | 2021 | 11-14 |
| Abdomen | Timor-Tritsch | 1989 | 7-12 |
|  | Van Zalen-Sprock | 1997 | 6-16 |
|  | Sase | [1999](https://doi.org/10.1515/jpm-2019-0222) | 12-39 |
|  | Sase | [2005](http://dx.doi.org/10.1136/hrt.77.1.68) | 12-39 |
|  | Gielchinsky | 2011 | 11-13 |
|  | Venkatesh | [2018](https://doi.org/10.1002/uog.6374) | 11-14 |
|  | Bogers | 2019 | 6-12^+6^ |
| Urogenital | Grannum | 1980 | >12 |
|  | Natsuyama | 1984 | 12-40 |
|  | Bronshtein | 1990 | 13-16 |
|  | Bronshtein | 1990 | 12-14 |
|  | Rosati | 1996 | 11-16 |
|  | Benoit | 1999 | 11-14 |
|  | Efrat | 1999 | 11-14 |
|  | Whitlow | 1999 | 11-14 |
|  | Lev-Toaff | 2000 | 10-24 |
|  | Mazza | 2001 | 11^+2^-13^+2^ |
|  | Michailidis | 2003 | 11-14 |
|  | Mazza | 2004 | Missing |
|  | Efrat | 2006 | 12-14 |
|  | Hsiao | 2008 | 11^+0^-13^+6^ |
|  | Chelli | 2009 | 11-14 |
|  | Youssef | 2011 | 11^+0^-13^+6^ |
|  | Behrendt | 2012 | 11^+0^-13^+6^ |
|  | Lubusky | 2012 | 12-15 |
|  | Manzanares | 2016 | 11^+0^-13^+6^ |
|  | Tudorache | 2016 | 11^+0^-13^+4^ |
|  | Arfi | 2016 | 11-14 |
|  | Bogers | 2018 | 9^+0^-12^+6^ |
|  | Sipahi | 2019 | 11^+0^-13^+6^ |
|  | Najdi | 2019 | 11^+0^-13^+6^ |
|  | Alfuraih | 2021 | 11-13^+6^ |
|  | Schaefer | 2023 | 4-11 |
| Other | Robinson | 1975 | 5-13^+3^ |
|  | Ghorashi | 1977 | 5-16 |
|  | Mantoni | 1979 | 6^+1^-9 |
|  | Sauerbrei | 1980 | 7-14 |
|  | Crooij | 1982 | 6^+2^-12^+3^ |
|  | Bree | 1989 | 4^+4^-7^+1^ |
|  | Cacciatore | 1990 | 4^+3^-5^+3^ |
|  | Jauniaux | 1991 | 5^+3^-12^+2^ |
|  | Daya | 1991 | >5^+1^ |
|  | Kurtz | 1992 | <13^+6^ |
|  | Haddow | 1992 | 5-8 |
|  | Steiner | 1994 | 5-11 |
|  | Hill | 1994 | 6^+1^-11 |
|  | Ohtsuka | 1995 | Missing |
|  | Coulam | 1996 | 4^+6^-8 |
|  | Stampone | 1996 | 5-12 |
|  | Blaas | 1998 | 7^+2^-11^+6^ |
|  | Babinszki | 1999 | >6 |
|  | Kupesic | 1999 | 5-12 |
|  | Muller | 2000 | 5-12 |
|  | Ghezzi | 2001 | 8-14^+6^ |
|  | Acharya | 2002 | 8^+2^-9^+6^ |
|  | Santolaya-Forgas | 2003 | 5-15 |
|  | Chama | 2005 | 5-11 |
|  | Lee | 2006 | 8-12 |
|  | Axt-Fliedner | 2006 | 11-14 |
|  | Verwoerd-Dikkeboom | 2008 | 6-14 |
|  | Odeh | 2008 | 6-13^+6^ |
|  | Goynumer | 2008 | 11-14^+6^ |
|  | Bottomley | 2009 | 4^+1^-11^+6^ |
|  | Rousian | 2009 | 6-11 |
|  | Rolo | 2009 | 7-10 |
|  | Borenstein | 2009 | 11-13^+6^ |
|  | Bagratee | 2009 | 6-12 |
|  | Nardozza | 2010 | 7-11 |
|  | Papaioannou | 2010 | 6-10, 11-13 |
|  | Sur | 2010 | 5-9^+5^ |
|  | Rôlo | 2010 | 7-10 |
|  | Kaur | 2011 | <12 |
|  | Araujo | 2011 | 7-10^+6^ |
|  | Rousian | 2011 | 5^+5^-12^+6^ |
|  | Rousian | 2011 | 6-13^+6^ |
|  | Köşüş | 2012 | 6^+6^-10^+6^ |
|  | Köşüş | 2012 | 6-10 |
|  | Tan | 2012 | 12-13^+6^ |
|  | Duran | 2014 | 11-14 |
|  | Bruns | 2015 | <13^+6^ |
|  | Narayan | 2015 | 11-13^+6^ |
|  | Batmaz | 2016 | 6^+6^-10^+4^ |
|  | Ali | 2018 | 6-10 |
|  | Odland Karlsen | 2019 | 8-12^+6^ |
|  | Suguna | 2019 | 6-9^+6^ |
|  | Derisbourg | 2019 | 11-14 |
|  | Detti | 2020 | 6-10 |
|  | Liao | 2020 | 6^+4^-13^+5^ |
|  | Marin | 2021 | 5^+5^-11^+6^ |
|  | Wang | 2021 | 6-8 |
| Muscoloskeletal | Exacoustos | 1991 | 13-40 |
|  | Zorzoli | 1994 | 9^+1^-15^+3^ |
|  | Van Zalen-Sprock | 1997 | 8-16 |
|  | Ververs | 1998 | 12-36 |
|  | Hata | 1998 | 12-40 |
|  | Merz | 2000 | 12-42 |
|  | Guariglia | 2002 | 10-16 |
|  | Kurjak | 2002 | 7-12 |
|  | De Biasio | 2002 | 9^+6^-14^+4^ |
|  | Rosati | 2002 | 11-16 |
|  | De Biasio | 2002 | 10^+5^-13^+5^ |
|  | Rosati | 2003 | 11-16 |
|  | Kanellopoulos | 2003 | 10-14 |
|  | Senat | 2003 | 11-14 |
|  | Cicero | 2003 | 11-14 |
|  | Viora | 2003 | 11-14 |
|  | Longo | 2004 | 11-14 |
|  | Rembouskos | 2004 | 11-14 |
|  | Cusick | 2004 | 11-20 |
|  | Kelekci | 2004 | 11-14 |
|  | Bekker | 2004 | 11-14 |
|  | Vignolo | 2005 | 10^+5^-13^+5^ |
|  | Faro | 2005 | 9-34 |
|  | Peralta | 2005 | 11-13^+6^ |
|  | Collado | 2005 | 11-14 |
|  | Faro | 2006 | 11-13^+6^ |
|  | Faro | 2006 | 11-13^+7^ |
|  | Leung | 2006 | 11-13^+6^ |
|  | Kozlowski | 2006 | 11-14 |
|  | Chen | 2006 | 11^+5^-14^+4^ |
|  | Moon | 2006 | 11-14 |
|  | Ramos-Corpas | 2006 | 11-14 |
|  | Chen | 2007 | 12^+0^-14^+6^ |
|  | Zalel | 2007 | 13-17 |
|  | Cossi | 2008 | 11-14^+6^ |
|  | Has | 2008 | 11-13^+6^ |
|  | Chen | 2009 | 11-13^+6^ |
|  | McLennan | 2009 | 11-13^+6^ |
|  | Sahota | 2009 | 11-13^+6^ |
|  | Casasbuenas | 2009 | 11-14 |
|  | Staboulidou | 2009 | 11^+2^-13^+6^ |
|  | Cheng | 2010 | 11-14 |
|  | Molina García | 2010 | 11-14^+4^ |
|  | Ozer | 2010 | 11-14 |
|  | Martinez-Ten | 2010 | 11-14 |
|  | Unsal | 2011 | 11-14 |
|  | Belics | 2011 | 11-20^+1^ |
|  | Thia | 2011 | 11-13^+6^ |
|  | Yayla | 2011 | 11-13^+6^ |
|  | Mihailovic | 2012 | 10^+1^-15^+5^ |
|  | Mǎrginean | 2012 | 11-13^+6^ |
|  | Pruksanusak | 2012 | 11-13^+6^ |
|  | Suwanrath | 2013 | 11-14 |
|  | Koo | 2014 | 11-13^+6^ |
|  | Baken | 2014 | 9-13^+2^ |
|  | Adiego | 2014 | 11-13 |
|  | Nanni | 2014 | 11-13^+6^ |
|  | Hermann | 2015 | 11-26 |
|  | Manegold-Brauer | 2015 | 11-14 |
|  | Seabra | 2015 | 13-30 |
|  | Liu | 2016 | 11^+2^-14^+1^ |
|  | Chaoui | 2016 | 12^+3^-12^+7^ |
|  | Henderson | 2016 | 12^+6^-35^+1^ |
|  | Liao | 2016 | 11^+0^-13^+6^ |
|  | Seabra | 2017 | 11-36 |
|  | Wong | 2017 | 10-16 |
|  | Kandasamy | 2018 | 11-14 |
|  | Lakshmy | [2019](https://doi.org/10.1002/uog.1775) | 11-14 |
|  | Achebe | 2019 | 13-41 |
|  | Bogers | 2019 | 6-12^+6^ |
|  | Ramkrishna | 2019 | 11-13^+6^ |
|  | Rao | 2019 | 12-39 |
|  | Manegold-Brauer | 2019 | 11^+2^-14^+1^ |
| Multiple organsystems | Timor-Tritsch | [1992](https://doi.org/10.7863/jum.2006.25.2.173) | 9-14 |
|  | Braithwaite | [1996](https://doi.org/10.7863/jum.2006.25.2.173) | 12-13^+6^ |
|  | Hata | [1997](https://doi.org/10.1016/j.echo.2017.03.017) | 8-13+6 |
|  | Hata | [1998](https://doi.org/10.7863/ultra.34.1.143) | 9-40 |
|  | Guariglia | 1998 | 11-16 |
|  | Rosati | [2000](https://doi.org/10.1016/0301-5629(93)90120-D) | 10^+1^-16^+0^ |
|  | Hull | [2001](https://doi.org/10.1002/uog.9068) | 12^+3^ |
|  | Michailidis | [2002](https://doi.org/10.7863/ultra.34.1.143) | 12-13^+6^ |
|  | Yonemoto | [2002](https://doi.org/10.1002/uog.9068) | 5-11 |
|  | Timor-Tritsch | 2004 | 11-12^+6^ |
|  | Souka | [2004](https://doi.org/10.7863/jum.2011.30.5.695) | 11-14 |
|  | Von Kaisenberg | [2005](https://doi.org/10.1016/0301-5629(93)90120-D) | 11-13^+6^ |
|  | Lombardi | 2007 | 12^+3^-14^+1^ |
|  | Wah | [2008](https://doi.org/10.1002/uog.6374) | 11-13^+6^ |
|  | Fauchon | 2008 | 11-13^+6^ |
|  | Ebrashy | [2010](https://doi.org/10.7863/jum.2006.25.2.173) | 13-14 |
|  | Bhaduri | [2010](https://doi.org/10.1016/j.echo.2017.03.017) | 12-13^+6^ |
|  | Antsaklis | [2011](https://doi.org/10.1016/j.echo.2017.03.017) | 11^+0^-13^+6^ |
|  | Borrell | [2011](https://doi.org/10.7863/jum.2011.30.5.695) | 11^+2^-13^+4^ |
|  | Luchi | 2012 | 11-13^+6^ |
|  | Adiego | [2012](https://doi.org/10.1002/uog.9068) | 11-13^+0^ |
|  | Hirides | [2012](https://doi.org/10.1002/uog.9068) | 11-14 |
|  | Abu-Rustum | [2012](https://doi.org/10.7863/jum.2006.25.2.173) | 11^+6^-13^+6^ |
|  | Lim | [2013](https://doi.org/10.7863/ultra.34.1.143) | 12-16^+6^ |
|  | Yagel | 2015 | 11^+3^-13^+2^ |
|  | Pooh | [2015](https://doi.org/10.7863/jum.2011.30.5.695) | 9-23 |
|  | Martinez-Ten | [2018](https://doi.org/10.1002/uog.3948) | 11^+0^-13^+6^ |
|  | McCormick | [2018](https://doi.org/10.1016/0301-5629(93)90120-D) | 12-14 |
|  | Toscano | [2019](https://doi.org/10.7863/jum.2011.30.5.695) | 13-15^+6^ |
|  | Sainz | 2020 | 11-13^+6^ |
|  | Sripilaipong | 2021 | 11-13^+6^ |
|  | Wiertsema | [2021](https://doi.org/10.7863/jum.2006.25.2.173) | 10^+6^-13^+2^ |
|  | Wiertsema | [2022](https://doi.org/10.7863/jum.2011.30.5.695) | 11^+4^-12^+7^ |
|  | Gireada | 2022 | 11-37 |
| **†**GA = Gestational age  * **References full citation details**  1. Altmann R, Scharnreitner I, Auer C, Hirtler L, Springer C, Falschlehner S, Arzt W. Visualization of the Third Ventricle, the Future Cavum Septi Pellucidi, and the Cavum Veli Interpositi at 11+3 to 13+6 Gestational Weeks on 3D Transvaginal Ultrasound Including Normative Data. Ultraschall in Der Medizin.  2. Liao CY, Peregrin-Alvarez I, Roman R, Morris J, Detti L. Early detection of velamentous cord insertion at the eighth week of gestation. Clinical Case Reports.  3. Toscano M, Grace D, Pressman EK, Thornburg LL. Does transvaginal ultrasound at 13-15 weeks improve anatomic survey completion rates in obese gravidas? Journal of Maternal-Fetal & Neonatal Medicine.  4. Robinson HP. "Gestation sac" volumes as determined by sonar in the first trimester of pregnancy. Br J Obstet Gynaecol. 1975;82(2):100-7.  5. Ghorashi B, Gottesfeld KR. The gray scale appearance of the normal pregnancy from 4 to 16 weeks of gestation. J Clin Ultrasound. 1977;5(3):195-201.  6. Mantoni M, Pedersen JF. Ultrasound visualization of the human yolk sac. J CLIN ULTRASOUND. 1979;7(6):459-60.  7. Grannum P, Bracken M, Silverman R, Hobbins JC. Assessment of fetal kidney size in normal gestation by comparison of ratio of kidney-circumference to abdominal circumference. AM J OBSTET GYNECOL. 1980;136(2):249-54.  8. Sauerbrei E, Cooperberg PL. Ultrasound demonstration of the normal fetal yolk sac. … of Clinical Ultrasound. 1980.  9. Crooij MJ, Westhuis M, Schoemaker J, Exalto N. Ultrasonographic measurement of the yolk sac. BR J OBSTET GYNAECOL. 1982;89(11):931-4.  10. Christ JE, Meininger MG. Ultrasound study of the nose and upper lip before birth. Ann Plast Surg. 1983;11(4):308-12.  11. Natsuyama E. Sonographic determination of fetal sex from twelve weeks of gestation. AM J OBSTET GYNECOL. 1984;149(7):748-57.  12. Bree RL, Edwards M, Bohm-Velez M, Beyler S, Roberts J, Mendelson EB. Transvaginal sonography in the evaluation of normal early pregnancy: Correlation with HCG level. AM J ROENTGENOL. 1989;153(1):75-9.  13. Kushnir U, Shalev J, Bronstein M, Bider D, Lipitz S, Nebel L, et al. Fetal intracranial anatomy in the first trimester of pregnancy: Transvaginal ultrasonographic evaluation. NEURORADIOLOGY. 1989;31(3):222-5.  14. Timor-Tritsch IE, Warren WB, Peisner DB, Pirrone E. First-trimester midgut herniation: A high-frequency transvaginal sonographic study. AM J OBSTET GYNECOL. 1989;161(3):831-3.  15. Bronshtein M, Kushnir O, Ben-Rafael Z, Shalev E, Nebel L, Mashiach S, Shalev J. Transvaginal sonographic measurement of fetal kidneys in the first trimester of pregnancy. J CLIN ULTRASOUND. 1990;18(4):299-301.  16. Bronshtein M, Rottem S, Yoffe N, Blumenfeld Z, Brandes JM. Early determination of fetal sex using transvaginal sonography: technique and pitfalls. J CLIN ULTRASOUND. 1990;18(4):302-6.  17. Cacciatore B, Tiitinen A, Stenman UH, Ylostalo P. Normal early pregnancy: Serum hCG levels and vaginal ultrasonography findings. BR J OBSTET GYNAECOL. 1990;97(10):899-903.  18. D'Amelio R, Giorlandino C, Masala L, Garofalo M, Martinelli M, Anelli G, Zichella L. Fetal echocardiography using transvaginal and transabdominal probes during the first period of pregnancy: a comparative study. Prenat Diagn. 1991;11(2):69-75.  19. Daya S, Woods S, Ward S, Lappalainen R, Caco C. Early pregnancy assessment with transvaginal ultrasound scanning. CAN MED ASSOC J. 1991;144(4):441-6.  20. Exacoustos C, Rosati P, Rizzo G, Arduini D. Ultrasound Measurements of Fetal Limb Bones. Ultrasound in Obstetrics & Gynecology. 1991;1(5):325-30.  21. Jauniaux E, Jurkovic D, Henriet Y, Rodesch F, Hustin J. Development of the secondary human yolk sac: Correlation of sonographic and anatomical features. HUM REPROD. 1991;6(8):1160-6.  22. Haddow JE, Holman MS, Palomaki GE. Can gestational dates routinely derived from very early ultrasound be used to interpret maternal serum alpha-fetoprotein measurements? PRENATAL DIAGN. 1992;12(1):65-8.  23. Johnson P, Sharland G, Maxwell D, Allan L. The role of transvaginal sonography in the early detection of congenital heart disease. Ultrasound Obstet Gynecol. 1992;2(4):248-51.  24. Kurtz AB, Needleman L, Pennell RG, Baltarowich O, Vilaro M, Goldberg BB. Can detection of the yolk sac in the first trimester be used to predict the outcome of pregnancy? A prospective sonographic study. AM J ROENTGENOL. 1992;158(4):843-7.  25. Timor-Tritsch IE, Monteagudo A, Peisner DB. High-frequency transvaginal sonographic examination for the potential malformation assessment of the 9-week to 14-week fetus. J CLIN ULTRASOUND. 1992;20(4):231-8.  26. Brezinka C, Stijnen T, Wladimiroff JW. Relationship between fetal pulmonary trunk and ductus arteriosus flow velocity waveforms in early normal pregnancy. ULTRASOUND MED BIOL. 1993;19(7):527-31.  27. Bronshtein M, Tzidony D, Dimant M, Hajos J, Jaeger M, Blumenfeld Z. Transvaginal Ultrasonographic Measurements of the Fetal Adrenal-Glands at 12 to 17 Weeks of Gestation. American Journal of Obstetrics and Gynecology. 1993;169(5):1205-10.  28. Achiron R, Weissman A, Rotstein Z, Lipitz S, Mashiach S, Hegesh J. Transvaginal Echocardiographic Examination of the Fetal Heart between 13-Weeks and 15-Weeks Gestation in a Low-Risk Population. Journal of Ultrasound in Medicine. 1994;13(10):783-9.  29. Blaas HG, Eik-Nes SH, Kiserud T, Hellevik LR. Early development of the forebrain and midbrain: a longitudinal ultrasound study from 7 to 12 weeks of gestation. Ultrasound Obstet Gynecol. 1994;4(3):183-92.  30. Hill LM, DiNofrio DM, Guzick D. Sonographic determination of first trimester umbilical cord length. J CLIN ULTRASOUND. 1994;22(7):435-8.  31. Steiner H, Gregg AR, Bogner G, Graf AH, Weiner CP, Staudach A. First trimester three-dimensional ultrasound volumetry of the gestational sac. ARCH GYNECOL OBSTET. 1994;255(4):165-70.  32. Zorzoli A, Kustermann A, Caravelli E, Corso FE, Fogliani R, Aimi G, Nicolini U. Measurements of fetal limb bones in early pregnancy. Ultrasound Obstet Gynecol. 1994;4(1):29-33.  33. Achiron R, Gottlieb Z, Yaron Y, Gabbay M, Gabbay U, Lipitz S, Mashiach S. The development of the fetal eye: In utero ultrasonographic measurements of the vitreous and lens. PRENATAL DIAGN. 1995;15(2):155-60.  34. Blaas HG, Eik-Nes SH, Kiserud T, Berg S, Angelsen B, Olstad B. Three-dimensional imaging of the brain cavities in human embryos. Ultrasound Obstet Gynecol. 1995;5(4):228-32.  35. Blaas HG, Eik-Nes SH, Kiserud T, Hellevik LR. Early development of the hindbrain: a longitudinal ultrasound study from 7 to 12 weeks of gestation. Ultrasound Obstet Gynecol. 1995;5(3):151-60.  36. Ohtsuka T, Taga M, Suzuki T, Minaguchi H. Relation between gestational sac diameter, crown-rump length, and maternal serum estradiol, progesterone, and prolactin levels in early pregnancy. ARCH GYNECOL OBSTET. 1995;256(1):5-8.  37. Braithwaite JM, Armstrong MA, Economides DL. Assessment of fetal anatomy at 12 to 13 weeks of gestation by transabdominal and transvaginal sonography. BR J OBSTET GYNAECOL. 1996;103(1):82-5.  38. Coulam CB, Britten S, Soenksen DM. Early (34-56 days from last menstrual period) ultrasonographic measurements in normal pregnancies. HUM REPROD. 1996;11(8):1771-4.  39. Rm Van ZS. First‐trimester sonographic detection of neurodevelopmental abnormalities in some single‐gene disorders. … in Affiliation With the …. 1996.  40. Rosati P, Guariglia L. Transvaginal sonographic assessment of the fetal urinary tract in early pregnancy. Ultrasound Obstet Gynecol. 1996;7(2):95-100.  41. Stampone C, Nicotra M, Muttinelli C, Cosmi EV. Transvaginal sonography of the yolk sac in normal and abnormal pregnancy. J CLIN ULTRASOUND. 1996;24(1):3-9.  42. Allan LD, Santos R, Pexieder T. Anatomical and echocardiographic correlates of normal cardiac morphology in the late first trimester fetus. HEART. 1997;77(1):68-72.  43. Hata T, Aoki S, Manabe A, Hata K, Miyazaki K. Three-dimensional ultrasonography in the first trimester of human pregnancy. HUM REPROD. 1997;12(8):1800-4.  44. Lam YH, Tang MHY. Prenatal diagnosis of haemoglobin Bart's disease by cordocentesis at 12-14 weeks' gestation. Prenatal Diagnosis. 1997;17(6):501-4.  45. Van Zalen-Sprock RM, Brons JTJ, Van Vugt JMG, Van Der Harten HJ, Van Geijn HP. Ultrasonographic and radiologic visualization of the developing embryonic skeleton. ULTRASOUND OBSTET GYNECOL. 1997;9(6):392-7.  46. Van Zalen-sprock RM, Van Vugt JMG, Van Geijn HP. First-trimester sonography of physiological midgut herniation and early diagnosis of omphalocele. PRENATAL DIAGN. 1997;17(6):511-8.  47. Blaas HG, Eik-Nes SH, Bremnes JB. The growth of the human embryo. A longitudinal biometric assessment from 7 to 12 weeks of gestation. Ultrasound Obstet Gynecol. 1998;12(5):346-54.  48. Bronshtein M, Zimmer EZ, Tzidony D, Hajos J, Jaeger M, Blazer S. Transvaginal sonographic measurement of fetal lingual width in early pregnancy. Prenatal Diagn. 1998;18(6):577-80.  49. Carvalho JS, Moscoso G, Ville Y. First-trimester transabdominal fetal echocardiography. Lancet. 1998;351(9108):1023-7.  50. Guariglia L, Rosati P. Transvaginal sonographic fetal biparietal diameter/kidney length ratio in early pregnancy as a screening tool for renal malformations. Fetal Diagn Ther. 1998;13(3):154-6.  51. Guariglia L, Rosati P. Early transvaginal measurement of transcerebellar diameter in Down syndrome. Fetal Diagn Ther. 1998;13(5):287-90.  52. Hata T, Aoki S, Akiyama M, Yanagihara T, Miyazaki K. Three-dimensional ultrasonographic assessment of fetal hands and feet. Ultrasound Obstet Gynecol. 1998;12(4):235-9.  53. Hata T, Aoki S, Hata K, Miyazaki K, Akahane M, Mochizuki T. Three-dimensional ultrasonographic assessments of fetal development. Obstet Gynecol. 1998;91(2):218-23.  54. Ververs IAP, Van Gelder-Hasker MR, De Vries JIP, Hopkins B, Van Geijn HP. Prenatal development of arm posture. Early Hum Dev. 1998;51(1):61-70.  55. Babinszki A, Mukherjee T, Kerenyi T, Berkowitz RL, Copperman AB. Diagnosing amnionicity at 6 weeks of pregnancy with transvaginal three-dimensional ultrasonography: case report. Fertil Steril. 1999;71(6):1161-4.  56. Benoit B. Early fetal gender determination. Ultrasound Obstet Gynecol. 1999;13(5):299-300.  57. Efrat Z, Akinfenwa OO, Nicolaides KH. First-trimester determination of fetal gender by ultrasound. Ultrasound Obstet Gynecol. 1999;13(5):305-7.  58. Leiva MC, Tolosa JE, Binotto CN, Weiner S, Huppert L, Denis AL, Huhta JC. Fetal cardiac development and hemodynamics in the first trimester. Ultrasound Obstet Gynecol. 1999;14(3):169-74.  59. Rosati P, Guariglia L. Cerebellar hypoplasia: Could it be a sonographic finding of abnormal fetal karyotype in early pregnancy? Fetal Diagn Ther. 1999;14(6):365-7.  60. Sase M, Tamura H, Ueda K, Kato H. Sonographic evaluation of antepartum development of fetal gastric motility. Ultrasound in Obstetrics & Gynecology. 1999;13(5):323-6.  61. Whitlow BJ, Lazanakis MS, Economides DL. The sonographic identification of fetal gender from 11 to 14 weeks of gestation. Ultrasound Obstet Gynecol. 1999;13(5):301-4.  62. Gembruch U, Shi CY, Smrcek JM. Biometry of the fetal heart between 10 and 17 weeks of gestation. Fetal Diagn Ther. 2000;15(1):20-31.  63. Lev-Toaff AS, Ozhan S, Pretorius D, Bega G, Kurtz AB, Kuhlman K. Three-dimensional multiplanar ultrasound for fetal gender assignment: Value of the mid-sagittal plane. Ultrasound Obstet Gynecol. 2000;16(4):345-50.  64. Merz E, Oberstein A, Wellek S. Age-related reference ranges for fetal foot length. Ultraschall Med. 2000;21(2):79-85.  65. Muller T, Sutterlin M, Pohls U, Dietl J. Transvaginal volumetry of first trimester gestational sac: a comparison of conventional with three-dimensional ultrasound. J Perinat Med. 2000;28(3):214-20.  66. Rosati P, Guariglia L, Bertuzzi A. Transvaginal assessment of fetal anatomy at 11 to 16 weeks of gestation in relation to fetal position. Fetal Diagn Ther. 2000;15(2):63-70.  67. Ghezzi F, Raio L, Di Naro E, Franchi M, Brhwiler H, D'Addario V, Schneider H. First-trimester sonographic umbilical cord diameter and the growth of the human embryo. Ultrasound Obstet Gynecol. 2001;18(4):348-51.  68. Hull AD, James G, Salerno CC, Nelson T, Pretorius DH. Three-dimensional ultrasonography and assessment of the first-trimester fetus. J Ultrasound Med. 2001;20(4):287-93.  69. Kupesic S, Kurjak A. Volume and vascularity of the yolk sac assessed by three-dimensional and power doppler ultrasound. Early Pregnancy. 2001;5(1):40-1.  70. Mazza V, Falcinelli C, Paganelli S, Contu G, Mantuano SM, Battafarano SD, et al. Sonographic early fetal gender assignment: A longitudinal study in pregnancies after in vitro fertilization. Ultrasound Obstet Gynecol. 2001;17(6):513-6.  71. Acharya G, Morgan H. First‐trimester, three‐dimensional transvaginal ultrasound volumetry in normal pregnancies and spontaneous miscarriages. … the International Society of Ultrasound …. 2002.  72. De Biasio P, Ginocchio G, Vignolo M, Ravera G, Venturini PL, Aicardi G. Spine length measurement in the first trimester of pregnancy. Prenat Diagn. 2002;22(9):818-22.  73. De Biasio P, Prefumo F, Lantieri PB, Venturini PL. Reference values for fetal limb biometry at 10-14 weeks of gestation. Ultrasound Obstet Gynecol. 2002;19(6):588-91.  74. Guariglia L, Rosati P. Early transvaginal biometry of fetal orbits: A cross-sectional study. Fetal Diagn Ther. 2002;17(1):42-7.  75. Haak MC, Twisk JWR, Van Vugt JMG. How successful is fetal echocardiographic examination in the first trimester of pregnancy? Ultrasound Obstet Gynecol. 2002;20(1):9-13.  76. Kurjak A, Vecek N, Hafner T, Bozek T, Funduk-Kurjak B, Ujevic B. Prenatal diagnosis: What does four-dimensional ultrasound add? J Perinat Med. 2002;30(1):57-62.  77. Michailidis GD, Papageorgiou P, Economides DL. Assessment of fetal anatomy in the first trimester using two- and three-dimensional ultrasound. Br J Radiol. 2002;75(891):215-9.  78. Radaelli T, Cetin I, Zamperini P, Ferrazzi E, Pardi G. Intrauterine growth of normal thyroid. Gynecol Endocrinol. 2002;16(6):427-30.  79. Rosati P, Bartolozzi F, Guariglia L. Reference values of fetal orbital measurements by transvaginal scan in early pregnancy. Prenat Diagn. 2002;22(10):851-5.  80. Yonemoto H, Yoshida K, Kinoshita K, Takeuchi H. Embryological evaluation of surface features of human embryos and early fetuses by 3-D ultrasound. J Obstet Gynaecol Res. 2002;28(4):211-6.  81. Cicero S, Dezerega V, Andrade E, Scheier M, Nicolaides KH. Learning curve for sonographic examination of the fetal nasal bone at 11-14 weeks. Ultrasound Obstet Gynecol. 2003;22(2):135-7.  82. Kanellopoulos V, Katsetos C, Economides DL. Examination of fetal nasal bone and repeatability of measurement in early pregnancy. Ultrasound Obstet Gynecol. 2003;22(2):131-4.  83. Michailidis GD, Papageorgiou P, Morris RW, Economides DL. The use of three-dimensional ultrasound for fetal gender determination in the first trimester. Br J Radiol. 2003;76(907):448-51.  84. Rosati P, Guariglia L. Early Transvaginal Fetal Orbital Measurements: A Screening Tool for Aneuploidy? J Ultrasound Med. 2003;22(11):1201-5.  85. Sacchini C, El-Sheikhah A, Cicero S, Rembouskos G, Nicolaides KH. Ear length in trisomy 21 fetuses at 11-14 weeks of gestation. Ultrasound Obstet Gynecol. 2003;22(5):460-3.  86. Santolaya-Forgas J, De Leon-Luis J, D'Ancona RL, Morgan J, Kauffman RP. Evolution of the amniotic sac and extracelomic space as seen by early ultrasound examination. Fetal Diagn Ther. 2003;18(4):262-9.  87. Senat MV, Bernard JP, Boulvain M, Ville Y. Intra- and interoperator variability in fetal nasal bone assessment at 11-14 weeks of gestation. Ultrasound Obstet Gynecol. 2003;22(2):138-41.  88. Viora E, Masturzo B, Errante G, Sciarrone A, Bastonero S, Campogrande M. Ultrasound evaluation of fetal nasal bone at 11 to 14 weeks in a consecutive series of 1906 fetuses. Prenat Diagn. 2003;23(10):784-7.  89. Bekker MN, Twisk JWR, Van Vugt JMG. Reproducibility of the fetal nasal bone length measurement. J Ultrasound Med. 2004;23(12):1613-8.  90. Carvalho JS, Moscoso G, Tekay A, Campbell S, Thilaganathan B, Shinebourne EA. Clinical impact of first and early second trimester fetal echocardiography on high risk pregnancies. Heart. 2004;90(8):921-6.  91. Cusick W, Provenzano J, Sullivan CA, Gallousis FM, Rodis JF. Fetal nasal bone length in euploid and aneuploid fetuses between 11 and 20 weeks' gestation: a prospective study. J Ultrasound Med. 2004;23(10):1327-33.  92. Huggon IC, Turan O, Allan LD. Doppler assessment of cardiac function at 11-14 weeks' gestation in fetuses with normal and increased nuchal translucency. Ultrasound in Obstetrics & Gynecology. 2004;24(4):390-8.  93. Kelekci S, Fehmi Yazicioǧlu H, Oguz S, Inan I, Yilmaz B, Sönmez S. Nasal bone measurement during the 1st trimester: Is it useful? Gynecol Obstet Invest. 2004;58(2):91-5.  94. Longo D, DeFigueiredo D, Cicero S, Sacchini C, Nicolaides KH. Femur and humerus length in trisomy 21 fetuses at 11-14 weeks of gestation. Ultrasound Obstet Gynecol. 2004;23(2):143-7.  95. Mazza V, Di Monte I, Pati M, Contu G, Ottolenghi C, Forabosco A, Volpe A. Sonographic biometrical range of external genitalia differentiation in the first trimester of pregnancy: Analysis of 2593 cases. Prenat Diagn. 2004;24(9):677-84.  96. Rembouskos G, Cicero S, Longo D, Vandecruys H, Nicolaides KH. Assessment of the fetal nasal bone at 11-14 weeks of gestation by three-dimensional ultrasound. Ultrasound Obstet Gynecol. 2004;23(3):232-6.  97. Singhakom N, Chawanpaiboon S, Titapant V. Reference centile charts for ratio of fetal transverse cerebellar diameter to abdominal circumference in a Thai population. J Med Assoc Thai. 2004;87 Suppl 3:S54-8.  98. Souka AP, Pilalis A, Kavalakis Y, Kosmas Y, Antsaklis P, Antsaklis A. Assessment of fetal anatomy at the 11-14-week ultrasound examination. Ultrasound Obstet Gynecol. 2004;24(7):730-4.  99. Timor-Tritsch IE, Bashiri A, Monteagudo A, Arslan AA. Qualified and trained sonographers in the US can perform early fetal anatomy scans between 11 and 14 weeks. Am J Obstet Gynecol. 2004;191(4):1247-52.  100. Chama CM, Marupa JY, Obed JY. The value of the secondary yolk sac in predicting pregnancy outcome. J Obstet Gynaecol. 2005;25(3):245-7.  101. Collado F, Bombard A, Li V, Julliard K, Aptekar L, Weiner Z. Ethnic variation of fetal nasal bone length between 11-14 weeks' gestation. Prenat Diagn. 2005;25(8):690-2.  102. Faro C, Benoit B, Wegrzyn P, Chaoui R, Nicolaides KH. Three-dimensional sonographic description of the fetal frontal bones and metopic suture. Ultrasound Obstet Gynecol. 2005;26(6):618-21.  103. Peralta CFA, Cavoretto P, Csapo B, Vandecruys H, Nicolaides KH. Assessment of lung area in normal fetuses at 12-32 weeks. Ultrasound Obstet Gynecol. 2005;26(7):718-24.  104. Peralta CFA, Falcon O, Wegrzyn P, Faro C, Nicolaides KH. Assessment of the gap between the fetal nasal bones at 11 to 13 + 6 weeks of gestation by three-dimensional ultrasound. Ultrasound Obstet Gynecol. 2005;25(5):464-7.  105. Sase M, Miwa I, Sumie M, Nakata M, Sugino N, Ross MG. Ontogeny of gastric emptying patterns in the human fetus. Journal of Maternal-Fetal & Neonatal Medicine. 2005;17(3):213-7.  106. Vignolo M, Ginocchio G, Parodi A, Torrisi C, Pistorio A, Venturini PL, et al. Fetal spine ossification: The gender and individual differences illustrated by ultrasonography. Ultrasound Med Biol. 2005;31(6):733-8.  107. von Kaisenberg CS, Kuhling-von Kaisenberg HK, Fritzer E, Schemm S, Meihold-Heerlein IM, Jonat W. Fetal transabdominal anatomy scanning using standard views at 11 to 14 weeks' gestation. American Journal of Obstetrics and Gynecology. 2005;192(2):535-42.  108. Axt-Fliedner R, Schwarze A, Kreiselmaier P, Krapp M, Smrcek J, Diedrich K. Umbilical cord diameter at 11-14 weeks of gestation: Relationship to nuchal translucency, ductus venous blood flow and chromosomal defects. Fetal Diagn Ther. 2006;21(4):390-5.  109. Chen M, Lee CP, Tang R, Chan B, Ou CQ, Tang MHY. First-trimester examination of fetal nasal bone in the Chinese population. Prenat Diagn. 2006;26(8):703-6.  110. Efrat Z, Perri T, Ramati E, Tugendreich D, Meizner I. Fetal gender assignment by first-trimester ultrasound. Ultrasound Obstet Gynecol. 2006;27(6):619-21.  111. Faro C, Wegrzyn P, Benoit B, Chaoui R, Nicolaides KH. Metopic suture in fetuses with holoprosencephaly at 11 + 0 to 13 + 6 weeks of gestation. Ultrasound Obstet Gynecol. 2006;27(2):162-6.  112. Faro C, Wegrzyn P, Benoit B, Chaoui R, Nicolaides KH. Metopic suture in fetuses with trisomy 21 at 11 + 0 to 13 + 6 weeks of gestation. Ultrasound Obstet Gynecol. 2006;27(3):286-9.  113. Kozlowski P, Knippel AJ, Froehlich S, Stressig R. Additional performance of nasal bone in first trimester screening: Nasal bone in first trimester screening. Ultraschall Med. 2006;27(4):336-9.  114. Lee W, Deters RL, McNie B, Powell M, Balasubramaniam M, Gonçalves LF, et al. Quantitative and morphological assessment of early gestational sacs using three-dimensional ultrasonography. Ultrasound Obstet Gynecol. 2006;28(3):255-60.  115. Leung TY, Chan LW, Leung TN, Fung TY, Sahota DS, Lau TK. First-trimester maternal serum level of pregnancy-associated plasma protein-A is an independent predictor of fetal maxillary bone length. Ultrasound Obstet Gynecol. 2006;27(1):9-12.  116. Moon MH, Cho JY, Lee YM, Lee YH, Yang JH, Kim MY, Park SH. Nasal bone length at 11-14 weeks of pregnancy in the Korean population. Prenat Diagn. 2006;26(6):524-7.  117. Peralta CFA, Cavoretto P, Csapo B, Falcon O, Nicolaides KH. Lung and heart volumes by three-dimensional ultrasound in normal fetuses at 12-32 weeks' gestation. Ultrasound Obstet Gynecol. 2006;27(2):128-33.  118. Ramos-Corpas D, Santiago JC, Montoya F. Ultrasonographic evaluation of fetal nasal bone in a low-risk population at 11-13 + 6 gestational weeks. Prenat Diagn. 2006;26(2):112-7.  119. Smrcek JM, Berg C, Geipel A, Fimmers R, Diedrich K, Gembruch U. Early fetal echocardiography: Heart biometry and visualization of cardiac structures between 10 and 15 weeks' gestation. J Ultrasound Med. 2006;25(2):173-82.  120. Vimpelli T, Huhtala H, Acharya G. Fetal echocardiography during routine first-trimester screening: a feasibility study in an unselected population. Prenat Diagn. 2006;26(5):475-82.  121. Borenstein M, Persico N, Kaihura C, Sonek J, Nicolaides KH. Frontomaxillary facial angle in chromosomally normal fetuses at 11 + 0 to 13 + 6 weeks. Ultrasound Obstet Gynecol. 2007;30(5):737-41.  122. Chen M, Chin PL, Yung HL, Chun QO, Tang MHY. First-trimester fetal limb biometry in Chinese population. Prenat Diagn. 2007;27(2):133-8.  123. Lombardi CM, Bellotti M, Fesslova V, Cappellini A. Fetal echocardiography at the time of the nuchal translucency scan. Ultrasound Obstet Gynecol. 2007;29(3):249-57.  124. Mittal P, Gonçalves LF, Kusanovic JP, Espinoza J, Lee W, Nien JK, et al. Objective evaluation of sylvian fissure development by multiplanar 3-dimensional ultrasonography. J Ultrasound Med. 2007;26(3):347-53.  125. Plasencia W, Dagklis T, Pachoumi C, Kolitsi E, Nicolaides KH. Frontomaxillary facial angle at 11 + 0 to 13 + 6 weeks: Effect of plane of acquisition. Ultrasound Obstet Gynecol. 2007;29(6):660-5.  126. Plasencia W, Dagklis T, Sotiriadis A, Borenstein M, Nicolaides KH. Frontomaxillary facial angle at 11 + 0 to 13 + 6 weeks' gestation - Reproducibility of measurements. Ultrasound Obstet Gynecol. 2007;29(1):18-21.  127. Zalel Y, Achiron R, Kivilevitch Z. Sonographic visualization of the middle phalanx of the fetal fifth digit between 13 and 17 weeks of gestation. Ultrasound Obstet Gynecol. 2007;30(3):263-5.  128. Cossi PS, Bussamra LCS, Araujo Jr E, Nardozza LMM, Moron AF. Ethnic variation and variability of fetal nasal bone length at 11-15 weeks of gestation in a Brazilian population: Preliminary results. Arch Gynecol Obstet. 2008;278(5):431-5.  129. Fauchon DEV, Benzie RJ, Wye DA, Cairns DR. What information on fetal anatomy can be provided by a single first-trimester transabdominal three-dimensional sweep? Ultrasound Obstet Gynecol. 2008;31(3):266-70.  130. Goynumer G, Ozdemir A, Wetherilt L, Durukan B, Yayla M. Umbilical cord thickness in the first and early second trimesters and perinatal outcome. J Perinat Med. 2008;36(6):523-6.  131. Has R, Kalelioglu I, Yuksel A, Ibrahimoglu L, Ermis H, Yildirim A. Fetal nasal bone assessment in first trimester down syndrome screening. Fetal Diagn Ther. 2008;24(1):61-6.  132. Hsiao CH, Wang HC, Hsieh CF, Hsu JJ. Fetal gender screening by ultrasound at 11 to 13+6 weeks. Acta Obstet Gynecol Scand. 2008;87(1):8-13.  133. Marques Carvalho SR, Mendes MC, Neto OBP, Berezowski AT. First trimester fetal echocardiography. Gynecol Obstet Invest. 2008;65(3):162-8.  134. McGahan MC, Ramos GA, Landry C, Wolfson T, Sowell BB, D'Agostini D, et al. Multislice Display of the Fetal Face Using 3-Dimensional Ultrasonography. Journal of Ultrasound in Medicine. 2008;27(11):1573-81.  135. Mi SK, Jeanty P, Turner C, Benoit B. Three-dimensional sonographic evaluations of embryonic brain development. J Ultrasound Med. 2008;27(1):119-24.  136. Molina FS, Faro C, Sotiriadis A, Dagklis T, Nicolaides KH. Heart stroke volume and cardiac output by four-dimensional ultrasound in normal fetuses. Ultrasound Obstet Gynecol. 2008;32(2):181-7.  137. Odeh M, Hirsh Y, Degani S, Grinin V, Ofir E, Bornstein J. Three-dimensional sonographic volumetry of the gestational sac and the amniotic sac in the first trimester. J Ultrasound Med. 2008;27(3):373-8.  138. Rôlo LC, Nardozza LMM, Araujo Jr E, Nowak PM, Moron AF. Yolk sac volume assessed by three-dimensional ultrasonography using the VOCAL method. Acta Obstet Gynecol Scand. 2008;87(5):499-502.  139. Tonni G, Azzoni D, Centini G, Ventura A. Correlation between 3D transvaginal first-trimester neuroimaging and embryonic development according to carnegie stadiation. J Diagn Med Sonogr. 2008;24(4):207-15.  140. Verwoerd-Dikkeboom CM, Koning AHJ, Hop WC, Rousian M, Van Der Spek PJ, Exalto N, Steegers EAP. Reliability of three-dimensional sonographic measurements in early pregnancy using virtual reality. Ultrasound Obstet Gynecol. 2008;32(7):910-6.  141. Viñals F, Ascenzo R, Naveas R, Huggon I, Giuliano A. Fetal echocardiography at 11 + 0 to 13 + 6 weeks using four-dimensional spatiotemporal image correlation telemedicine via an Internet link: A pilot study. Ultrasound Obstet Gynecol. 2008;31(6):633-8.  142. Wah YM, Chan LW, Leung TY, Fung TY, Lau TK. How true is a 'true' midsagittal section? Ultrasound Obstet Gynecol. 2008;32(7):855-9.  143. Bagratee JS, Regan L, Khullar V, Connolly C, Moodley J. Reference intervals of gestational sac, yolk sac and embryo volumes using three-dimensional ultrasound. Ultrasound Obstet Gynecol. 2009;34(5):503-9.  144. Bennasar M, Martinez JM, Olivella A, Del Rio M, Gomez O, Figueras F, et al. Feasibility and accuracy of fetal echocardiography using four-dimensional spatiotemporal image correlation technology before 16 weeks' gestation. Ultrasound in Obstetrics & Gynecology. 2009;33(6):645-51.  145. Borenstein M, Perez GA, Garcia FM, Romero M, Anderica JR. Gestational sac volume: comparison between SonoAVC and VOCAL measurements at 11+0 to 13+6 weeks of gestation. Ultrasound in Obstetrics & Gynecology. 2009;34(5):510-4.  146. Bottomley C, Daemen A, Mukri F, Papageorghiou AT, Kirk E, Pexsters A, et al. Assessing first trimester growth: The influence of ethnic background and maternal age. Hum Reprod. 2009;24(2):284-90.  147. Casasbuenas A, Wong AE, Sepulveda W. First-trimester nasal bone length in a normal Latin American population. Prenat Diagn. 2009;29(2):108-12.  148. Chaoui R, Benoit B, Mitkowska-Wozniak H, Heling KS, Nicolaides KH. Assessment of intracranial translucency (IT) in the detection of spina bifida at the 11-13-week scan. Ultrasound Obstet Gynecol. 2009;34(3):249-52.  149. Chelli D, Methni A, Dimassi K, Boudaya F, Sfar E, Zouaoui B, et al. Fetal sex assignment by first trimester ultrasound: A Tunisian experience. Prenat Diagn. 2009;29(12):1145-8.  150. Chen M, Hui FW, Tak YL, Tak YF, Lin WC, Sahota DS, et al. First trimester measurements of nasal bone length using three-dimensional ultrasound. Prenat Diagn. 2009;29(8):766-70.  151. Hata T, Dai SY, Kanenishi K, Tanaka H. Three-dimensional volume-rendered imaging of embryonic brain vesicles using inversion mode. J Obstet Gynaecol Res. 2009;35(2):258-61.  152. McLennan A, Schluter PJ, Pincham V, Hyett J. First-trimester fetal nasal bone audit: evaluation of a novel method of image assessment. Ultrasound Obstet Gynecol. 2009;34(6):623-8.  153. Pistorius L, Stoutenbeek P, Visser GHA. First trimester neurosonoembryology with automated follicle tracking: Preliminary findings. J Matern -Fetal Neonatal Med. 2009;22(10):949-51.  154. Rolo LC, Nardozza LMM, Araujo Jr E, Nowak PM, Moron AF. Gestational sac volume by 3D-sonography at 7-10 weeks of pregnancy using the VOCAL method. Arch Gynecol Obstet. 2009;279(6):821-7.  155. Rousian M, Verwoerd-Dikkeboom CM, Koning AHJ, Hop WC, Van Der Spek PJ, Exalto N, Steegers EAP. Early pregnancy volume measurements: Validation of ultrasound techniques and new perspectives. BJOG Int J Obstet Gynaecol. 2009;116(2):278-85.  156. Sahota DS, Leung TY, Chan LW, Law LW, Fung TY, Chan OK, Lau TK. First-trimester fetal nasal bone length in an ethnic Chinese population. Ultrasound Obstet Gynecol. 2009;34(1):33-7.  157. Staboulidou I, Wüstemann M, Vaske B, Scharf A, Hillemanns P, Schmidt P. Interobserver variability of the measurement of fetal nasal bone length between 11 + 0 and 13 + 6 gestation weeks among experienced and inexperienced sonographers. Ultraschall Med. 2009;30(1):42-6.  158. Turan S, Turan OM, Ty-Torredes K, Harman CR, Baschat AA. Standardization of the first-trimester fetal cardiac examination using spatiotemporal image correlation with tomographic ultrasound and color Doppler imaging. Ultrasound Obstet Gynecol. 2009;33(6):652-6.  159. Uittenbogaard LB, Haak MC, Spreeuwenberg MD, Van Vugt JMG. Fetal cardiac function assessed with four-dimensional ultrasound imaging using spatiotemporal image correlation. Ultrasound Obstet Gynecol. 2009;33(3):272-81.  160. Alphonse J, Cox J, Clarke J, Schluter PJ, McLennan A. Comparison of frontomaxillary facial angles using both 2D and 3D ultrasound at 11+0 to 13+6 weeks of gestation. Fetal Diagn Ther. 2010;28(1):14-21.  161. Bhaduri M, Fong K, Toi A, Tomlinson G, Okun N. Fetal anatomic survey using three-dimensional ultrasound in conjunction with first-trimester nuchal translucency screening. Prenat Diagn. 2010;30(3):267-73.  162. Cheng PJ, Huang SY, Shaw SW, Chueh HY, Soong YK. Evaluation of fetal spine biometry between 11 and 14 weeks of gestation. Ultrasound Med Biol. 2010;36(7):1060-5.  163. Ebrashy A, El Kateb A, Momtaz M, El Sheikhah A, Aboulghar MM, Ibrahim M, Saad M. 13-14-week fetal anatomy scan: a 5-year prospective study. Ultrasound Obstet Gynecol. 2010;35(3):292-6.  164. Jeon SR, Choi HM, Roh YH, Kim YH, Son GH, Nam KH, et al. Frontomaxillary facial angle measurements in euploid Korean fetuses at 11 weeks' to 13 weeks 6 days' gestation. J Ultrasound Med. 2010;29(11):1565-71.  165. MacHado Nardozza LM, Rolo LC, Araujo Jr E, Nowak PM, Filho JB, Moron AF. Comparison of gestational sac volume by 3D-sonography using planimetric, virtual organ computer-aided analysis and extended imaging virtual organ computer-aided analysis methods between 7 and 11 weeks of pregnancy. Acta Obstet Gynecol Scand. 2010;89(3):328-34.  166. Martinez-Ten P, Adiego B, Perez-Pedregosa J, Illescas T, Wong AE, Sepulveda W. First-trimester assessment of the nasal bones using the retronasal triangle view: a 3-dimensional sonographic study. J Ultrasound Med. 2010;29(11):1555-61.  167. Molina García FS, Carrillo Badillo MP, Zaragoza García EA, Fernández De Santos AG, Montoya Ventoso F. Analysis of secondary ultrasound markers in the first trimester before chorionic villus sampling. Prenat Diagn. 2010;30(12-13):1117-20.  168. Ozer A, Ozaksit G, Kanat-Pektas M, Ozer S. First trimester examination of fetal nasal bone in the Turkish population. J Obstet Gynaecol Res. 2010;36(4):739-44.  169. Papaioannou GI, Syngelaki A, Poon LCY, Ross JA, Nicolaides KH. Normal ranges of embryonic length, embryonic heart rate, gestational sac diameter and yolk sac diameter at 6-10 weeks. Fetal Diagn Ther. 2010;28(4):207-19.  170. Rozmus-Warcholinska W, Wloch A, Acharya G, Cnota W, Czuba B, Sodowski K, Skrzypulec V. Reference values for variables of fetal cardiocirculatory dynamics at 11-14 weeks of gestation. Ultrasound Obstet Gynecol. 2010;35(5):540-7.  171. Sepulveda W, Wong AE, Martinez-Ten P, Perez-Pedregosa J. Retronasal triangle: a sonographic landmark for the screening of cleft palate in the first trimester. Ultrasound Obstet Gynecol. 2010;35(1):7-13.  172. Sinkovskaya E, Horton S, Berkley EM, Cooper JK, Indika S, Abuhamad A. Defining the fetal cardiac axis between 11 + 0 and 14 + 6 weeks of gestation: experience with 100 consecutive pregnancies. Ultrasound Obstet Gynecol. 2010;36(6):676-81.  173. Sur SD, Jayaprakasan K, Jones NW, Clewes J, Winter B, Cash N, et al. A Novel Technique for the Semi-Automated Measurement of Embryo Volume: An Intraobserver Reliability Study. Ultrasound in Medicine and Biology. 2010;36(5):719-25.  174. Vicario R, Pirollo LMA, Angelis CD, Narcisi M, Pietropolli A, Piccione E. Frontonasal facial angle in chromosomally normal fetuses at 11+0 to 13+6 weeks. J Obstet Gynaecol Res. 2010;36(6):1179-84.  175. Xiong Y, Wah YM, Chan LW, Leung TY, Fung TY, Lau TK. Real-time three-dimensional ultrasound with Live xPlane imaging assists first-trimester acquisition of a true midsagittal section. Ultrasound Obstet Gynecol. 2010;36(2):136-40.  176. Yang X, Chen M, Wang HF, Leung TY, Borenstein M, Nicolaides K, et al. Learning curve in measurement of fetal frontomaxillary facial angle at 11-13 weeks of gestation. Ultrasound Obstet Gynecol. 2010;35(5):530-4.  177. Abu-Rustum RS, Ziade MF, Abu-Rustum SE. Learning curve and factors influencing the feasibility of performing fetal echocardiography at the time of the first-trimester scan. J Ultrasound Med. 2011;30(5):695-700.  178. Antsaklis A, Daskalakis G, Theodora M, Hiridis P, Komita O, Blanas K, Anastasakis E. Assessment of nuchal translucency thickness and the fetal anatomy in the first trimester of pregnancy by two- and three-dimensional ultrasonography: A pilot study. J Perinat Med. 2011;39(2):185-93.  179. Araujo Jr E, Nardozza LMM, Rolo LC, Haratz KK, Moron AF. Assessment of yolk sac volume by 3D-sonography using the XI VOCAL method from 7 to 10 + 6 weeks of pregnancy. Arch Gynecol Obstet. 2011;283(SUPPL. 1):S1-S4.  180. Belics Z, Fekete T, Beke A, Szabó I. Prenatal ultrasonographic measurement of the fetal iliac angle during the first and second trimester of pregnancy. Prenat Diagn. 2011;31(4):351-5.  181. Borrell A, Santolaya-Forgas J, Horbaczewski C, Henry RD, Dunn-Albanese L, Robinson JN. Is the starting section for 3D volume acquisition in the first trimester relevant in the post hoc analysis of aneuploidy screening markers and fetal anatomy? Prenat Diagn. 2011;31(13):1305-10.  182. Brestak M, Sonek J, Tomek V, McKenna D, Calda P. Shortening fraction of the right ventricle: a comparison between euploid and trisomy 21 fetuses at week 11 to week 13 and 6 days of gestation. Prenat Diagn. 2011;31(8):760-4.  183. Chaoui R, Benoit B, Heling KS, Kagan KO, Pietzsch V, Sarut Lopez A, et al. Prospective detection of open spina bifida at 11-13 weeks by assessing intracranial translucency and posterior brain. Ultrasound Obstet Gynecol. 2011;38(6):722-6.  184. Chen M, Wang HF, Leung TY, Sahota DS, Borenstein M, Nicolaides K, et al. Frontomaxillary facial angle at 11 + 0 to 13 + 6 weeks in Chinese population. J Matern Fetal Neonatal Med. 2011;24(3):498-501.  185. Egle D, Strobl I, Weiskopf-Schwendinger V, Grubinger E, Kraxner F, Mutz-Dehbalaie IS, et al. Appearance of the fetal posterior fossa at 11 + 3 to 13 + 6 gestational weeks on transabdominal ultrasound examination. Ultrasound Obstet Gynecol. 2011;38(6):620-4.  186. Finn M, Sutton D, Atkinson S, Ransome K, Sujenthiran P, Ditcham V, et al. The aqueduct of Sylvius: a sonographic landmark for neural tube defects in the first trimester. Ultrasound Obstet Gynecol. 2011;38(6):640-5.  187. Gielchinsky Y, Zvanca M, Minekawa R, Persico N, Nicolaides KH. Liver volume in trisomy 21 and euploid fetuses at 11 to 13 weeks. Prenat Diagn. 2011;31(1):28-32.  188. Ginsberg NA, Cohen L, Dungan JS, Concialdi S, Mangers K, Shulman LP. 3-D ultrasound of the fetal ear and fetal autosomal trisomies: A pilot study of a new screening protocol. Prenat Diagn. 2011;31(3):311-4.  189. Jadaon JE, Haddad S, Mukary M, Ben-Shlomo I, Ben-Ami M. Evaluation of normal fetal atrio-ventricular septum dimensions during pregnancy. Prenat Diagn. 2011;31(2):167-70.  190. Kaur A, Kaur A. Transvaginal ultrasonography in first trimester of pregnancy and its comparison with transabdominal ultrasonography. J Pharm Bioallied Sci. 2011;3(3):329-38.  191. Krapp M, Ludwig A, Axt-Fliedner R, Kreiselmaier P. First trimester fetal echocardiography: which planes and defects can be displayed during the daily routine in a prenatal medicine unit? Ultraschall Med. 2011;32(4):362-6.  192. Lachmann R, Chaoui R, Moratalla J, Picciarelli G, Nicolaides KH. Posterior brain in fetuses with open spina bifida at 11 to 13 weeks. Prenat Diagn. 2011;31(1):103-6.  193. Mangione R, Lelong N, Fontanges M, Amat S, Rosenblatt J, Khoshnood B, Jouannic JM. Visualization of intracranial translucency at the 11-13-week scan is improved after specific training. Ultrasound Obstet Gynecol. 2011;38(6):635-9.  194. Rousian M, Koning AH, Hop WC, van der Spek PJ, Exalto N, Steegers EA. Gestational sac fluid volume measurements in virtual reality. Ultrasound Obstet Gynecol. 2011;38(5):524-9.  195. Rousian M, Verwoerd-Dikkeboom CM, Koning AHJ, Hop WC, van der Spek PJ, Steegers EAP, Exalto N. First trimester umbilical cord and vitelline duct measurements using virtual reality. Early Hum Dev. 2011;87(2):77-82.  196. Scheier M, Lachmann R, Pětroš M, Nicolaides KH. Three-dimensional sonography of the posterior fossa in fetuses with open spina bifida at 11-13 weeks' gestation. Ultrasound Obstet Gynecol. 2011;38(6):625-9.  197. Solt I, Acuna JG, Adeniji BA, Mirocha J, Kim MJ, Rotmensch S. First-trimester visualization of the fourth ventricle in fetuses with and without spina bifida. J Ultrasound Med. 2011;30(12):1643-7.  198. Thia EW, Wei X, Tan DT, Lai XH, Zhang XJ, Oo SY, Yeo GS. Evaluation of an objective method of image assessment for first-trimester nasal bone. Ultrasound Obstet Gynecol. 2011;38(5):533-7.  199. Unsal N, Ozat M, Kanat-Pektas M, Gungor T, Danisman N. The significance of fetal maxillary length in the first trimester screening for trisomy 21. Arch Gynecol Obstet. 2011;283(6):1199-205.  200. Yayla M, Ergin RN, Göynümer G. Normative values of fetal nasal bone lengths of Turkish singleton pregnancies in the first trimester. J Turkish German Gynecol Assoc Artemis. 2011;12(4):225-8.  201. Youssef A, Arcangeli T, Radico D, Contro E, Guasina F, Bellussi F, et al. Accuracy of fetal gender determination in the first trimester using three-dimensional ultrasound. Ultrasound Obstet Gynecol. 2011;37(5):557-61.  202. Yuan Y, Leung KY, Ouyang YS, Yang F, Tang MHY, Chau AKT, Dai Q. Simultaneous real-time imaging of four-chamber and left ventricular outflow tract views using xPlane imaging capability of a matrix array probe. Ultrasound in Obstetrics & Gynecology. 2011;37(3):302-9.  203. Abu-Rustum RS, Ziade MF, Abu-Rustum SE. Defining the spatial relationships between eight anatomic planes in the 11+6 to 13+6 weeks fetus: A pilot study. Prenat Diagn. 2012;32(9):875-82.  204. Adiego B, Illescas T, Martinez-Ten P, Bermejo C, Perez-Pedregosa J, Wong AE, Sepulveda W. Intracranial translucency at 11-13 weeks of gestation: Prospective evaluation and reproducibility of measurements. Prenat Diagn. 2012;32(3):259-63.  205. Beamon CJ, Stuebe AM, Wolfe HM. Factors influencing visualization of the intracranial translucency during first-trimester screening for aneuploidy. Am J Perinatol. 2012;29(7):503-7.  206. Behrendt N, Foy P, Center J, Durnwald CP. Influence of maternal body mass index and gestational age on accuracy of first trimester gender assignment. J Matern -Fetal Neonatal Med. 2012;25(3):253-6.  207. Chen M, Chen H, Yang X, Wang HF, Yeung Leung T, Singh Sahota D, et al. Normal range of intracranial translucency (IT) assessed by three-dimensional ultrasound at 11 + 0 to 13 + 6 weeks in a Chinese population. J Matern -Fetal Neonatal Med. 2012;25(5):489-92.  208. Ergin RN, Yayla M. The nomogram of intracranial translucency in the first trimester in singletons. J Turkish German Gynecol Assoc Artemis. 2012;13(3):153-6.  209. Gindes L, Matsui H, Achiron R, Mohun T, Ho SY, Gardiner H. Comparison of ex-vivo high-resolution episcopic microscopy with in-vivo four-dimensional high-resolution transvaginal sonography of the first-trimester fetal heart. Ultrasound Obstet Gynecol. 2012;39(2):196-202.  210. Hirides P, Daskalakis G, Papantoniou N, Anastasakis E, Theodora M, Blanas K, Antsaklis A. A comparison between 2-D and off-line 3-D ultrasound in the assessment of fetal anatomies: A prospective analysis of 1007 cases. J Neonatal-Perinat Med. 2012;5(4):347-55.  211. Kavalakis I, Souka AP, Pilalis A, Papastefanou I, Kassanos D. Assessment of the posterior brain at 11-14 weeks for the prediction of open neural tube defects. Prenat Diagn. 2012;32(12):1143-6.  212. Köşüş A, Köşüş N, Turhan NO. Are the first trimester volumetric measurements with two dimensional ultrasonography satisfactory. J Matern -Fetal Neonatal Med. 2012;25(7):1117-21.  213. Köşüş N, Köşüş A, Turhan NO. First trimester volumetric measurements: Relation with hormone levels and fetal heart rate. Arch Gynecol Obstet. 2012;286(2):365-72.  214. Lee MY, Won HS, Jeong BD, Hyun MK, Lee HY, Shim JY, et al. Measurement of intracranial translucency using three-dimensional ultrasound and Volume IT™. Prenat Diagn. 2012;32(5):472-5.  215. Loureiro T, Ushakov F, Maiz N, Montenegro N, Nicolaides KH. Lateral ventricles in fetuses with aneuploidies at 11-13 weeks' gestation. Ultrasound Obstet Gynecol. 2012;40(3):282-7.  216. Loureiro T, Ushakov F, Montenegro N, Gielchinsky Y, Nicolaides KH. Cerebral ventricular system in fetuses with open spina bifida at 11-13 weeks' gestation. Ultrasound Obstet Gynecol. 2012;39(6):620-4.  217. Lubusky M, Studnickova M, Skrivanek A, Vomackova K, Prochazka M. Ultrasound evaluation of fetal gender at 12-14 weeks. Biomed Pap. 2012;156(4):324-9.  218. Luchi C, Schifano M, Sacchini C, Nanini C, Sceusa F, Capriello P, Genazzani AR. Detailed fetal anatomy assessment in the first trimester at 11, 12 and 13 weeks of gestation. J Matern -Fetal Neonatal Med. 2012;25(6):675-8.  219. Mǎrginean C, Brînzaniuc K, Voidǎzan S, Mǎrginean CO. Nasal bone assessment by 3D versus 2D ultrasound in the 11-13 gestational weeks screening. Gineco ro. 2012;8(4):165-70.  220. Mihailovic T, Stimec BV, Terzic M, Dmitrovic A, Micic J. The absence of the vomer in the first and early second trimester of pregnancy - a new marker of trisomy 21 and trisomy 13. Ultraschall Med. 2012;33(7):E68-74.  221. Pruksanusak N, Suwanrath C, Kor-Anantakul O, Suntharasaj T, Hanprasertpong T, Pranpnus S, Geater AF. A thai reference for normal fetal nasal bone length at 11-13(+6) weeks gestation. Gynecol Obstet Invest. 2012;73(3):211-6.  222. Rembouskos G, Passamonti U, De Robertis V, Tempesta A, Campobasso G, Volpe G, et al. Aberrant right subclavian artery (ARSA) in unselected population at first and second trimester ultrasonography. Prenat Diagn. 2012;32(10):968-75.  223. Sepulveda W, Cafici D, Bartholomew J, Wong AE, Martinez-Ten P. First-trimester assessment of the fetal palate: a novel application of the Volume NT algorithm. J Ultrasound Med. 2012;31(9):1443-8.  224. Tan S, Pektas MK, Ozcan AS, Akçay Y, Ozat M, Arslan H. Frequency of a persistent yolk sac and its relationship with the gestational outcome. J Ultrasound Med. 2012;31(5):697-702.  225. Votino C, Kacem Y, Dobrescu O, Dessy H, Cos T, Foulon W, Jani J. Use of a high-frequency linear transducer and MTI filtered color flow mapping in the assessment of fetal heart anatomy at the routine 11 to 13 + 6-week scan: a randomized trial. Ultrasound Obstet Gynecol. 2012;39(2):145-51.  226. Abu-Rustum RS, Ziade MF, Abu-Rustum SE. Reference values for the right and left fetal choroid plexus at 11 to 13 weeks: an early sign of "developmental" laterality? J Ultrasound Med. 2013;32(9):1623-9.  227. Alphonse J, Cox J, Clarke JL, Robinson CL, Schluter PJ, McLennan A. Frontomaxillary facial angle measurement at 11-14 weeks: assessment of interobserver and intraobserver agreement in 2D and 3D imaging. Fetal Diagn Ther. 2013;34(2):90-5.  228. Garcia-Posada R, Eixarch E, Sanz M, Puerto B, Figueras F, Borrell A. Cisterna magna width at 11-13 weeks in the detection of posterior fossa anomalies. Ultrasound Obstet Gynecol. 2013;41(5):515-20.  229. Hsiao CH, Liu WL, Chen RC, Cheng BJ, Tseng YJ, Chu WC. The fetal frontomaxillary facial angle in normal and trisomy 21 ultrasounds at 11-13(+6) weeks of gestation: findings among the ethnic Chinese compared with Caucasian. Prenat Diagn. 2013;33(8):711-5.  230. Leibovitz Z, Egenburg S, Bronshtein M, Shapiro I, Tepper R, Malinger G, Ohel G. Sonographic imaging of fetal tympanic rings. Ultrasound Obstet Gynecol. 2013;42(5):536-44.  231. Liberty G, Boldes R, Shen O, Shaul C, Cohen SM, Yagel S. The fetal larynx and pharynx: structure and development on two- and three-dimensional ultrasound. Ultrasound Obstet Gynecol. 2013;42(2):140-8.  232. Lim J, Whittle WL, Lee YM, Ryan G, Van Mieghem T. Early anatomy ultrasound in women at increased risk of fetal anomalies. Prenat Diagn. 2013;33(9):863-8.  233. Lima AIF, Araujo Júnior E, Martins WP, Nardozza LMM, Moron AF, Pares DBS. Assessment of the fetal heart at 12-14 weeks of pregnancy using B-mode, color doppler, and spatiotemporal image correlation via abdominal and vaginal ultrasonography. Pediatr Cardiol. 2013;34(7):1577-82.  234. Marginean C, Marginean CO, Brinzaniuc K, Voidazan S, Pop TS. Alveolar ridge at 11-13 weeks screening - A 3D ultrasound study. Gineco eu. 2013;9(2):93-7.  235. McBrien A, Howley L, Yamamoto Y, Hutchinson D, Hirose A, Sekar P, et al. Changes in fetal cardiac axis between 8 and 15 weeks' gestation. Ultrasound Obstet Gynecol. 2013;42(6):653-8.  236. Panigassi AP, Araujo Junior E, Nardozza LM, Moron AF, Pares DB. Fetal frontomaxillary facial angle between 11 and 13 + 6 weeks of gestation in a Brazilian population: influence of different races. J Matern Fetal Neonatal Med. 2013;26(11):1116-20.  237. Pares DBS, Lima AIF, Araujo Júnior E, Nardozza LMM, Martins WP, Moron AF. Fetal heart assessment in the first trimester of pregnancy: Influence of crown-rump length and maternal body mass index. Braz J Cardiovasc Surg. 2013;28(4):477-81.  238. Rousian M, Hop WC, Koning AHJ, Van Der Spek PJ, Exalto N, Steegers EAP. First trimester brain ventricle fluid and embryonic volumes measured by three-dimensional ultrasound with the use of I-Space virtual reality. Hum Reprod. 2013;28(5):1181-9.  239. Suwanrath C, Pruksanusak N, Kor-anantakul O, Suntharasaj T, Hanprasertpong T, Pranpanus S. Reliability of fetal nasal bone length measurement at 11-14 weeks of gestation. BMC Pregnancy Childbirth. 2013;13.  240. Tudorache S, Cara M, Iliescu DG, Novac L, Cernea N. First trimester two- and four-dimensional cardiac scan: intra- and interobserver agreement, comparison between methods and benefits of color Doppler technique. Ultrasound Obstet Gynecol. 2013;42(6):659-68.  241. Votino C, Cos T, Abu-Rustum R, Dahman Saidi S, Gallo V, Dobrescu O, et al. Use of spatiotemporal image correlation at 11-14 weeks' gestation. Ultrasound Obstet Gynecol. 2013;42(6):669-78.  242. Yazdi B, Riefler P, Fischmüller K, Sonek J, Hoopmann M, Kagan KO. The frontal space measurement in euploid and aneuploid pregnancies at 11-13weeks' gestation. Prenat Diagn. 2013;33(12):1124-30.  243. Zajicek M, Achiron R, Weisz B, Shrim A, Gindes L. Sonographic assessment of fetal secondary palate between 12 and 16 weeks of gestation using three-dimensional ultrasound. Prenat Diagn. 2013;33(13):1256-9.  244. Adiego B, Martinez-Ten P, Illescas T, Bermejo C, Sepulveda W. First-trimester assessment of nasal bone using retronasal triangle view: a prospective study. Ultrasound Obstet Gynecol. 2014;43(3):272-6.  245. Alphonse J, Cox J, Clarke JL, Schluter PJ, McLennan A. Frontomaxillary facial angle measurement in trisomy 21 and euploid fetuses: Two-and three-dimensional assessment during routine first trimester screening. Fetal Diagn Ther. 2014;36(3):183-9.  246. Baken L, Benoit B, Koning AHJ, Willemsen SP, van der Spek PJ, Steegers-Theunissen RPM, et al. First-trimester hand measurements in euploid and aneuploid human fetuses using virtual reality. Prenat Diagn. 2014;34(10):961-9.  247. Brestak M, Calda P, McKenna D, Sonek J. Comparison of right ventricular measurements and SFRV in fetuses with and without tricuspid regurgitation at 11+0 and 13+6 weeks' gestation. J Matern -Fetal Neonatal Med. 2014;27(15):1531-4.  248. Duran M, Kosus A, Kosus N, Turhan NO. Relation between serum PAPP-A level and umbilical cord thickness during first trimester of pregnancy. J Matern Fetal Neonatal Med. 2014;27(4):385-7.  249. Fong KW, Dengler J, Toi A, Menezes RJ, Karimzad Y, Okun N. Prospective study of intracranial translucency and the posterior brain in normal fetuses at the 11- to 13-week scan. J Ultrasound Med. 2014;33(8):1373-9.  250. Gijtenbeek M, Bogers H, Groenenberg IAL, Exalto N, Willemsen SP, Steegers EAP, et al. First trimester size charts of embryonic brain structures. Hum Reprod. 2014;29(2):201-7.  251. Koo FH, Wang PH, Wang HI, Wu YC, Juang CM, Chen YJ, et al. Measurement of fetal maxillary and mandibular angles for first-trimester prenatal screening among Taiwanese women. J Chin Med Assoc. 2014;77(8):430-2.  252. Nanni M, Maroni E, Bevini M, Cucchi G, Pignotti E, Pilu G, et al. The usefulness of volume NT software in measuring the fetal nasal bone at 11 to 13+6weeks of gestation. Prenat Diagn. 2014;34(5):500-4.  253. Orlandi E, Rossi C, Perino A, Musicò G, Orlandi F. Simplified first-trimester fetal cardiac screening (four chamber view and ventricular outflow tracts) in a low-risk population. Prenat Diagn. 2014;34(6):558-63.  254. Persico N, Fabietti I, Baffero GM, Fedele L, Nicolaides KH. Fetal right ventricular contraction and relaxation times at 11-13 weeks' gestation on speckle tracking imaging. Ultrasound Obstet Gynecol. 2014;43(3):284-90.  255. Shyu IL, Yang MJ, Wang HI, Wang PH, Chang CM, Juang CM, et al. Fetal maxillary and mandibular length in normal pregnancies from 11 weeks' to 13+6 weeks' gestation: A Taiwanese study. Taiwanese J Obstet Gynecol. 2014;53(1):53-6.  256. Yuksel MA, Arisoy R, Erdogdu E, Imamoglu M, Yayla M, Sen C. Relationship between first trimester visualization of the intracranial translucency and spina bifida. Arch Gynecol Obstet. 2014;291(3):513-8.  257. Boitor-Borza D, Kovacs T, Stamatian F. Ganglionic eminence within the early developing brain visualized by 3D transvaginal ultrasound. Med Ultrasonography. 2015;17(3):289-94.  258. Bruns RF, Menegatti CM, Martins WP, Júnior EA. Applicability of pocket ultrasound during the first trimester of pregnancy. Med Ultrasonography. 2015;17(3):284-8.  259. Chaoui R, Orosz G, Heling KS, Sarut-Lopez A, Nicolaides KH. Maxillary gap at 11-13 weeks' gestation: marker of cleft lip and palate. Ultrasound Obstet Gynecol. 2015;46(6):665-9.  260. Gindes L, Malach S, Weisz B, Achiron R, Leibovitz Z, Weissmann-Brenner A. Measuring the perimeter and area of the Sylvian fissure in fetal brain during normal pregnancies using 3-dimensional ultrasound. Prenat Diagn. 2015;35(11):1097-105.  261. Hermann NV, Darvann TA, Sundberg K, Kreiborg S, Joergensen C. Maxillary length in 11- to 26-week-old normal fetuses studied by 3D ultrasound. Prenat Diagn. 2015;35(6):571-6.  262. Hsiao CH, Cheng PJ, Shaw SWS, Tseng YJ, Chen RC, Chu WC. The BS/BSOB ratio in aneuploidy fetuses at 11-13 weeks gestation. Fetal Diagn Ther. 2015;37(4):321-6.  263. Kappou D, Papastefanou I, Pilalis A, Kavalakis I, Kassanos D, Souka AP. Towards detecting open spina bifida in the first trimester: The examination of the posterior brain. Fetal Diagn Ther. 2015;37(4):294-300.  264. Liu M, Liu Y, Li ZH, Yu D. Screening for Fetal Spina Bifida Aperta by the Ultrasound and Intracranial Translucency Examinations at 11-13(+6) Weeks of Gestation. Cell Biochemistry and Biophysics. 2015;72(2):439-41.  265. Manegold-Brauer G, Bourdil L, Berg C, Schoetzau A, Gembruch U, Geipel A. Prenasal thickness to nasal bone length ratio in normal and trisomy 21 fetuses at 11-14 weeks of gestation. Prenat Diagn. 2015;35(11):1079-84.  266. Narayan R, Saaid R, Pedersen L, Hyett J. Ultrasound Assessment of Umbilical Cord Morphology in the First Trimester: A Feasibility Study. Fetal Diagn Ther. 2015;38(3):212-7.  267. Nemescu D, Onofriescu M. Factors affecting the feasibility of routine first-trimester fetal echocardiography. J Ultrasound Med. 2015;34(1):161-6.  268. Pooh RK, Kurjak A. Novel application of three-dimensional HDlive imaging in prenatal diagnosis from the first trimester. J Perinat Med. 2015;43(2):147-58.  269. Seabra M, Felino A, Nogueira R, Valente F, Braga AC, Vaz P. Prenatal ultrasound and postmortem histologic evaluation of tooth germs: an observational, transversal study. Head & Face Medicine. 2015;11.  270. Sinkovskaya ES, Chaoui R, Karl K, Andreeva E, Zhuchenko L, Abuhamad AZ. Fetal cardiac axis and congenital heart defects in early gestation. Obstet Gynecol. 2015;125(2):453-60.  271. Weissmann-Brenner A, Zemet R, Kivilevitch Z, Zalel Y. Sonographic Evaluation of the Fetal Thymus Using the Thy-Box Technique Between 13 and 16 Weeks' Gestation. J Ultrasound Med. 2015;34(12):2263-8.  272. Yagel S, Cohen SM, Porat S, Daum H, Lipschuetz M, Amsalem H, et al. Detailed transabdominal fetal anatomic scanning in the late first trimester versus the early second trimester of pregnancy. J Ultrasound Med. 2015;34(1):143-9.  273. Zhen L, Pan M, Han J, Yang X, Ou YM, Liao C, Li DZ. Non-invasive prenatal detection of haemoglobin Bart's disease by cardiothoracic ratio during the first trimester. Eur J Obstet Gynecol Reprod Biol. 2015;193:92-5.  274. Adekola H, Soto E, Dai J, Puder K, Abramowicz JS. Fetal cardiac axis in non-anomalous pregnancies: does fetal gender or maternal body mass index (BMI) matter? Journal of Maternal-Fetal & Neonatal Medicine. 2016;29(13):2125-30.  275. Altmann R, Scharnreitner I, Scheier T, Mayer R, Arzt W, Scheier M. Sonoembryology of the fetal posterior fossa at 11 + 3 to 13 + 6 gestational weeks on three-dimensional transvaginal ultrasound. Prenat Diagn. 2016;36(8):731-7.  276. Arfi A, Cohen J, Canlorbe G, Bendifallah S, Thomassin-Naggara I, Darai E, et al. First-trimester determination of fetal gender by ultrasound: Measurement of the ano-genital distance. 2016;203:177-81.  277. Batmaz G, Aksoy A, Aydin S, Ozcan P, Dane C, Dane B. The early pregnancy volume measurements in predicting pregnancy outcome. Clin Exp Obstet Gynecol. 2016;43(2):241-4.  278. Borgelt JMA, Möllers M, Falkenberg MK, Amler S, Klockenbusch W, Schmitz R. Assessment of first-trimester thymus size and correlation with maternal diseases and fetal outcome. Acta Obstet Gynecol Scand. 2016;95(2):210-6.  279. Dmitrovic A, Jeremic K, Babic UM, Perovic M, Mihailovic T, Opric D, et al. Early fetal heart ultrasonography as additional indicator for chromosomopathies. Clin Exp Obstet Gynecol. 2016;43(2):245-9.  280. Henderson P, Desai IP, Pettit K, Benke S, Brouha SS, Romine LE, et al. Evaluation of Fetal First and Second Cervical Vertebrae: Normal or Abnormal? J Ultrasound Med. 2016;35(3):527-36.  281. Hoopmann M, Sonek J, Esser T, Bilardo CM, Wagner P, Abele H, Kagan KO. Frontal space distance in facial clefts and retrognathia at 11-13 weeks' gestation. Ultrasound Obstet Gynecol. 2016;48(2):171-6.  282. Krishnan A, Pike JI, McCarter R, Fulgium AL, Wilson E, Donofrio MT, Sable CA. Predictive Models for Normal Fetal Cardiac Structures. Journal of the American Society of Echocardiography. 2016;29(12):1197-206.  283. Liao YM, Li SL, Luo GY, Wen HX, Ouyang SY, Chen CY, et al. Routine screening for fetal limb abnormalities in the first trimester. Prenat Diagn. 2016;36(2):117-26.  284. Liu W, Qu S, Wang M, Xu W, Zhang G, Zhang C. Maxilla-nasion-mandible (MNM) angle: an indicator to assess fetal facial profile in first-trimester of pregnancy. Springerplus. 2016;5(1):1335.  285. Manegold-Brauer G, Oseledchyk A, Floeck A, Berg C, Gembruch U, Geipel A. Approach to the sonographic evaluation of fetal ventriculomegaly at 11 to 14 weeks gestation. BMC Pregnancy Childbirth. 2016;16(1).  286. Manzanares S, Benítez A, Naveiro-Fuentes M, López-Criado MS, Sánchez-Gila M. Accuracy of fetal sex determination on ultrasound examination in the first trimester of pregnancy. J Clin Ultrasound. 2016;44(5):272-7.  287. Molina-Giraldo S, Pérez-Olivo JL, Arias JL, Acuña E, Alfonso D, Arreaza M, Leal MB. Normal Intracranial Translucency Values During the First Trimester of Gestation in a Latin American Population. J Ultrasound Med. 2016;35(10):2231-6.  288. Nanthakomon T, Pongrojpaw D, Chanthasenannont A, Somprasit C, Tanprasertkul C, Suwannarurk K. Nomogram of intracranial translucency in the first trimester ultrasound of pregnant women at Thammasat university hospital. J Med Assoc Thailand. 2016;99:S32-S6.  289. Peixoto AB, Caldas TM, Lasmar LA, Martins WP, Pares DB, Araujo E. Reference range for the fetal intracranial translucency measurement between 11 and 14+2 weeks of gestation in a Brazilian population. J Matern -Fetal Neonatal Med. 2016;29(16):2588-91.  290. Pranpanus S, Kor-Anantakul O, Suntharasaj T, Suwanrath C, Leetanaporn R, Hanprasertpong T, Pruksanusak N. Frontomaxillary facial angle in chromosomally normal Thai foetuses at 11 to 13 weeks 6 days' gestation. J Obstet Gynaecol. 2016;36(1):53-7.  291. Tudorache S, Cara M, Iliescu DG, Stoica A, Simionescu C, Novac LV, Cernea D. Fetal Kidneys Ultrasound Appearance in the First Trimester - Clinical Significance and Limits of Counseling. Curr Health Sci J. 2016;42(1):19-30.  292. Yu R, Li S, Luo G, Wen H, Ouyang S, Chen C, Yuan Y. First-Trimester Echocardiographic Features and Perinatal Outcomes in Fetuses With Congenital Absence of the Aortic Valve. J Ultrasound Med. 2016;35(4):739-45.  293. De Robertis V, Rembouskos G, Fanelli T, Volpe G, Muto B, Volpe P. The three-vessel and trachea view (3VTV) in the first trimester of pregnancy: an additional tool in screening for congenital heart defects (CHD) in an unselected population. Prenat Diagn. 2017;37(7):693-8.  294. Hutchinson D, McBrien A, Howley L, Yamamoto Y, Sekar P, Motan T, et al. First-Trimester Fetal Echocardiography: Identification of Cardiac Structures for Screening from 6 to 13 Weeks’ Gestational Age. J Am Soc Echocardiogr. 2017;30(8):763-72.  295. Koning IV, Dudink J, Groenenberg IAL, Willemsen SP, Reiss IKM, Steegers-Theunissen RPM. Prenatal cerebellar growth trajectories and the impact of periconceptional maternal and fetal factors. Hum Reprod. 2017;32(6):1230-7.  296. Lakshmy SR, Deepa S, Rose N, Mookan S, Agnees J. First-Trimester Sonographic Evaluation of Palatine Clefts: A Novel Diagnostic Approach. J Ultrasound Med. 2017;36(7):1397-414.  297. Lakshmy SR, Jain B, Rose N. Role of HDLive in Imaging the Fetal Heart. J Ultrasound Med. 2017;36(6):1267-78.  298. Ponmozhi G, Manikandan K, Gopish D, Rameshkumar J, Suresh S, Suresh I, Seshadri S. Beyond Open Neural Tube Defects: Sagittal Landmarks at 11-14 Weeks in the Prediction of Second Trimester Posterior Fossa Abnormalities. Journal of Fetal Medicine. 2017;4(1):31-6.  299. Quarello E, Lafouge A, Fries N, Salomon LJ. Basic heart examination: feasibility study of first-trimester systematic simplified fetal echocardiography. Ultrasound Obstet Gynecol. 2017;49(2):224-30.  300. Seabra M, Vaz P, Valente F, Braga A, Felino A. Two-dimensional identification of fetal tooth germs. Cleft Palate-Craniofac J. 2017;54(2):166-9.  301. Shah H, Al-Memar M, de Bakker B, Fourie H, Lees C, Bourne T. The first-trimester fetal central nervous system: a novel ultrasonographic perspective. Am J Obstet Gynecol. 2017;217(2):220-1.  302. Teegala ML, Vinayak DG. Intracranial translucency as a sonographic marker for detecting open spina bifida at 11-13+6 weeks scan: Our experience. Indian J Radiol Imaging. 2017;27(4):427-31.  303. Wanapirak C, Sirichotiyakul S, Luewan S, Srisupundit K, Tongprasert F, Tongsong T. Appearance of Abnormal Cardiothoracic Ratio of Fetuses with Hemoglobin Bart's Disease: Life Table Analysis. Ultraschall Med. 2017;38(5):544-8.  304. Wong HS. A revisit of the fetal foot length and fetal measurements in early pregnancy sonography. Int J Womens Health. 2017;9:199-204.  305. Yang SH, An HS, Lee JS, Kim C. Normal intracranial BS/BSOB ratio values in the first trimester of single gestations with live fetuses in a Korean population. Med Ultrasonography. 2017;19(2):190-4.  306. Ali S, Byanyima RK, Ononge S, Ictho J, Nyamwiza J, Loro ELE, et al. Measurement error of mean sac diameter and crown-rump length among pregnant women at Mulago hospital, Uganda. BMC Pregnancy Childbirth. 2018;18(1):129.  307. Altmann R, Specht C, Scharnreitner I, Schertler C, Mayer R, Arzt W, Scheier M. Reference Ranges for Transvaginal Examined Fossa Posterior Structures in Fetuses from 45 to 84 mm Crown-Rump Length. Gynecol Obstet Invest. 2018;83(4):375-80.  308. Bogers H, Rifouna MS, Koning AHJ, Husen-Ebbinge M, Go ATJI, van der Spek PJ, et al. Accuracy of fetal sex determination in the first trimester of pregnancy using 3D virtual reality ultrasound. J Clin Ultrasound. 2018;46(4):241-6.  309. Kandasamy S, Raj SP. Nasal Bone Assessment and Credibility of Visualization Between 11 and 14 Weeks: Experience in a Tertiary Fetal Medicine Center. Journal of Fetal Medicine. 2018;5(1):13-6.  310. Kayaalti ES, Kumbasar S, Şik BA. The effectiveness of extended fetal echocardiography in evaluating fetal cardiac morphology. Clin Exp Obstet Gynecol. 2018;45(2):182-6.  311. Kose S, Altunyurt S, Keskinoglu P. A prospective study on fetal posterior cranial fossa assessment for early detection of open spina bifida at 11–13 weeks. Congenit Anomal. 2018;58(1):4-9.  312. Martinez-Ten P, Illescas T, Adiego B, Estevez M, Bermejo C, Wong AE, Sepulveda W. Non-visualization of choroid plexus of fourth ventricle as first-trimester predictor of posterior fossa anomalies and chromosomal defects. Ultrasound Obstet Gynecol. 2018;51(2):199-207.  313. McCormick A, Anaya H, Mehendale R, Fogg L, Roche M, Abramowicz J. Comparison of first trimester transvaginal anatomy scan to the second trimester structural survey in normal weight and obese populations: a pilot study. J Matern -Fetal Neonatal Med. 2018;31(17):2319-24.  314. Parisi F, Rousian M, Koning IV, Willemsen SP, de Vries JHM, Steegers EAP, Steegers-Theunissen RPM. Periconceptional maternal dairy-rich dietary pattern is associated with prenatal cerebellar growth. PLoS ONE. 2018;13(5):e0197901.  315. Venkatesh P. A Simple and Easy Technique for Imaging the Fetal Esophagus in the First, Second, and Third Trimesters Using the Transverse Section of the Esophagus in the Area Behind the Heart as a Reference Point. J Ultrasound Med. 2018;37(12):2863-72.  316. Wojtowicz A, Wojtowicz W, Jurek J, Huras H. Evaluation of the fetal palate at 11 to 13 (+6) weeks of gestation based on an analysis of static ultrasound images using modern IT techniques. Prenatal Diagnosis. 2018;38(6):414-21.  317. Achebe CC, Adesiyun OAM, Aremu IB, Orewole OT, Aremu SK, Bakare A, et al. Determination of gestational age by tibial length using ultrasound in a Nigerian Tertiary Hospital. Int J Biomed Sci. 2019;15(4):104-11.  318. Asoglu MR, Yao R, Seger L, Turan OM, Turan S. Applicability of Standardized Early Fetal Heart Examination in the Obese Population. J Ultrasound Med. 2019;38(5):1269-77.  319. Bogers H, Baken L, Cohen-Overbeek TE, Koning AHJ, Willemsen SP, Van Der Spek PJ, et al. Evaluation of first-trimester physiological midgut herniation using three-dimensional ultrasound. Fetal Diagn Ther. 2019;45(5):332-8.  320. Bogers H, Rifouna MS, Cohen-Overbeek TE, Koning AHJ, Willemsen SP, van der Spek PJ, et al. First trimester physiological development of the fetal foot position using three-dimensional ultrasound in virtual reality. J Obstet Gynaecol Res. 2019;45(2):280-8.  321. Comanescu MC, Capitanescu RG, Comanescu AC, Cernea N, Popa A, Barbu EM, Albulescu DM. First Trimester Neurosonogram-Our Experience. Curr Health Sci J. 2019;45(2):167-73.  322. Derisbourg S, Boulay A, Lamy C, Barlow P, Van Rysselberge M, Thomas D, et al. First trimester ultrasound prediction of velamentous cord insertions: a prospective study. J Matern -Fetal Neonatal Med. 2019.  323. Ebrashy A, Aboulghar M, Elhodiby M, El-Dessouky SH, Elsirgany S, Gaafar HM, et al. Fetal heart examination at the time of 13 weeks scan: A 5 years' prospective study. J Perinat Med. 2019;47(8):871-8.  324. Ferreira C, Rouxinol-Dias AL, Loureiro T, Nicolaides K. Subarachnoid space diameter in chromosomally abnormal fetuses at 11–13 weeks’ gestation. J Matern -Fetal Neonatal Med. 2019;32(12):2079-83.  325. García Fernández S, Arenas Ramirez J, Otero Chouza MT, Rodriguez-Vijande Alonso B, Llaneza Coto Á P. Early fetal ultrasound screening for major congenital heart defects without Doppler. 2019;233:93-7.  326. Garcia-Rodriguez R, Garcia-Delgado R, Romero-Requejo A, Medina-Castellano M, Garcia-Hernandez JA, Gonzalez-Martin JM, Sepulveda W. First-trimester cystic posterior fossa: reference ranges, associated findings, and pregnancy outcomes. J Matern -Fetal Neonatal Med. 2019.  327. Lakshmy SR, Rose N, Masilamani P, Umapathy S, Ziyaulla T. Role of TUI in First Trimester Evaluation of Palate. Journal of Fetal Medicine. 2019;6(3):113-21.  328. Majeed A, Abuhamad A, Romary L, Sinkovskaya E. Can Ultrasound in Early Gestation Improve Visualization of Fetal Cardiac Structures in Obese Pregnant Women? J Ultrasound Med. 2019;38(8):2057-63.  329. Manegold-Brauer G, Maymon R, Shor S, Cuckle H, Gembruch U, Geipel A. Down's syndrome screening at 11-14 weeks' gestation using prenasal thickness and nasal bone length. Arch Gynecol Obstet. 2019;299(4):939-45.  330. Najdi N, Safi F, Hashemi-Dizaji S, Sahraian G, Jand Y. First trimester determination of fetal gender by ultrasonographic measurement of anogenital distance: A cross-sectional study. Int J Reprod Biomed. 2019;17(1):51-6.  331. Odland Karlsen H, Johnsen SL, Rasmussen S, Trae G, Reistad HMT, Kiserud T. The human yolk sac size reflects involvement in embryonic and fetal growth regulation. Acta Obstet Gynecol Scand. 2019;98(2):176-82.  332. Pauta M, Grande M, Borobio V, Illanes C, Soler A, Nogué L, Borrell A. Is Cisterna Magna Width a Useful First-Trimester Marker of Aneuploidy? Fetal Diagn Ther. 2019.  333. Ramkrishna J, Araujo Júnior E, Peixoto AB, Da Silva Costa F, Meagher S. Maxillo–occipital line: a sonographic marker for screening of open spina bifida in the first trimester of pregnancy. J Matern -Fetal Neonatal Med. 2019;32(24):4073-9.  334. Rao R, Gornbein J, Afshar Y, Platt LD, DeVore GR, Krakow D. A new biometric: In utero growth curves for metacarpal and phalangeal lengths reveal an embryonic patterning ratio. Prenat Diagn. 2019;39(3):200-8.  335. Sipahi M, Tokgöz VY, Alanya Tosun Ş. An appropriate way to predict fetal gender at first trimester: anogenital distance. J Matern -Fetal Neonatal Med. 2019;32(12):2012-6.  336. Suguna B, Sukanya K. Yolk sac size & shape as predictors of first trimester pregnancy outcome: A prospective observational study. J Gynecol Obstet Hum Reprod. 2019;48(3):159-64.  337. Zheng MM, Tang HR, Zhang Y, Ru T, Li J, Xu BY, et al. Contribution of the Fetal Cardiac Axis and V-Sign Angle in First-Trimester Screening for Major Cardiac Defects. J Ultrasound Med. 2019;38(5):1179-87.  338. Chaoui R, Benoit B, Entezami M, Frenzel W, Heling KS, Ladendorf B, et al. Ratio of fetal choroid plexus to head size: simple sonographic marker of open spina bifida at 11-13 weeks' gestation. Ultrasound Obstet Gynecol. 2020;55(1):81-6.  339. Detti L, Francillon L, Christiansen ME, Peregrin-Alvarez I, Goeske PJ, Bursac Z, Roman RA. Early pregnancy ultrasound measurements and prediction of first trimester pregnancy loss: A logistic model. Sci Rep. 2020;10(1):1545.  340. Jung YJ, Lee BR, Kim GJ. Efficacy of fetal cardiac axis evaluation in the first trimester as a screening tool for congenital heart defect or aneuploidy. Obstet gynecol sci. 2020;63(3):278-85.  341. Kamel H, Yehia A. First trimester fetal echocardiography limitations and its expected clinical values. Egypt Heart J. 2020;72(1).  342. Sainz JA, Gutierrez L, García-Mejido J, Ramos Z, Bonomi MJ, Fernández-Palacín A, Aquise A. Early fetal morphological evaluation (11–13 + 6 weeks) accomplished exclusively by transabdominal imaging and following routine midtrimester fetal ultrasound scan recommendations. Since when can it be performed? J Matern -Fetal Neonatal Med. 2020;33(7):1140-50.  343. Alfuraih AM, Alotaiby SA, Alsaadi MJ, Bukhari HA, Aldhebaib AM, Mohtasib RS. Predictive value and reference ranges of anogenital distance for determining fetal gender in the first trimester: A retrospective cohort study. Saudi Med J. 2021;42(10):1057-64.  344. Basaran OE, Guvendag Guven ES, Guven S. First trimester fetal thymus volume may predict preeclampsia. Pregnancy Hypertens. 2021;26:116-20.  345. Eric Ozdemir M, Demirci O, Ayvaci Tasan H, Ohanoglu K, Akalin M. The importance of first trimester screening of cranial posterior fossa in predicting posterior fossa malformations which may be identified in the following weeks of gestation. J Clin Ultrasound. 2021;49(9):958-62.  346. Garcia Delgado R, Garcia Rodriguez R, Ortega Cardenes I, Gonzalez Martin JM, De Luis Alvarado M, Segura Gonzalez J, et al. Feasibility and Accuracy of Early Fetal Echocardiography Performed at 13+0-13+6 Weeks in a Population with Low and High Body Mass Index: a Prospective Study. Reprod Sci. 2021;28(8):2270-7.  347. Ji C, Jiang X, Yin L, Deng X, Yang Z, Pan Q, et al. Ultrasonographic study of fetal facial profile markers during the first trimester. BMC Pregnancy Childbirth. 2021;21(1):324.  348. Lian XH, Xu ZH, Zheng LP, Zhu ZX, Ejiwale T, Kumar A, et al. Reference range of fetal thorax using two-dimensional and three-dimensional ultrasound VOCAL technique and application in fetal thoracic malformations. Bmc Medical Imaging. 2021;21(1).  349. Marin M, Patru CL, Manolea MM, Novac L, Dijmarescu AL, Boldeanu MV, et al. Can Ultrasound Analysis of the Yolk Sac be a Predictor of Pregnancy Outcome? Curr Health Sci J. 2021;47(4):547-52.  350. Ramirez Zegarra R, Volpe N, Bertelli E, Amorelli GM, Ferraro L, Schera GBL, et al. Three-Dimensional Sonographic Evaluation of the Position of the Fetal Conus Medullaris at First Trimester. Fetal Diagn Ther. 2021;48(6):464-71.  351. Sripilaipong S, Panburana P, Wattanayingcharoenchai R, Tangshewinsirikul C. Feasibility and learning curve of performing first trimester fetal anatomy screening among operators with varying experience using the protocol of the International Society of Ultrasound in Obstetrics and Gynecology (ISUOG). J Matern Fetal Neonatal Med. 2021:1-7.  352. Sternfeld AR, Betzer T, Tamir A, Mizrachi Y, Assa S, Bar J, Gindes L. Can fetal echocardiographic measurements of the left ventricular outflow tract angle detect fetuses with conotruncal cardiac anomalies? Diagn. 2021;11(7).  353. Volpe P, De Robertis V, Volpe G, Boito S, Fanelli T, Olivieri C, et al. Position of the choroid plexus of the fourth ventricle in first- and second-trimester fetuses: a novel approach to early diagnosis of cystic posterior fossa anomalies. Ultrasound Obstet Gynecol. 2021;58(4):568-75.  354. Wang Y, Li T, Zhang L, Li J, Zou B, Singh BK. The Clinical Value of 3D Ultrasonic Measurement of the Ratio of Gestational Sac Volume to Embryo Volume in IoT-Based Prediction of Pregnancy Outcome. J healthc eng. 2021;2021:6421025.  355. Wiertsema CJ, Erkamp JS, Mulders A, Steegers EAP, Duijts L, Koning AHJ, et al. First trimester fetal proportion volumetric measurements using a Virtual Reality approach. Prenat Diagn. 2021;41(7):868-76.  356. Ye B, Wu Y, Chen J, Yang Y, Niu J, Wang H, et al. The diagnostic value of the early extended fetal heart examination at 13 to 14 weeks gestational age in a high-risk population. Transl pediatr. 2021;10(11):2907-20.  357. Feng L, Sun L, Wang J, Sun C, Lu L, Zhang Z, et al. Qualitative and quantitative study of fetal posterior fossa during the first trimester in a Chinese population. BMC Pregnancy Childbirth. 2022;22(1):760.  358. Gireada R, Socolov D, Mihalceanu E, Matasariu R, Ursache A, Akad M, et al. The Additional Role of the 3-Vessels and Trachea View in Screening for Congenital Heart Disease. Medicina-Lithuania. 2022;58(2).  359. Hata T, Koyanagi A, Kawahara T, Konishi M, Takayoshi R, Miyagi Y, Miyake T. HDlive Flow Silhouette with spatiotemporal image correlation for assessment of fetal cardiac structures at 12 to 14 + 6 weeks of gestation. J Perinat Med. 2022;50(3):313-8.  360. Husen SC, Kemper N, Go A, Willemsen SP, Rousian M, Steegers-Theunissen RPM. Periconceptional maternal folate status and the impact on embryonic head and brain structures: the Rotterdam Periconceptional Cohort. Reprod Biomed Online. 2022;44(3):515-23.  361. Ji X, Han Z, Wu Y, Zhang S. Study on normal reference value of fetal facial profile markers during the first trimester of pregnancy. Transl pediatr. 2022;11(1):99-107.  362. Li H, Zhu Z. Ultrasonographic study of fetal mandibular markers during the first trimester in a Chinese population. J Obstet Gynaecol Res. 2022;48(2):333-9.  363. Lin DM, Zhu YX, Tan Y, Huang YJ, Yuan K, Liu WF, Xu ZF. Feasibility of Transabdominal Real-time CDFI and HDFI Techniques for Fetal Pulmonary Vein Display in the First Trimester. Curr Med Sci. 2022;42(3):635-41.  364. Wiertsema CJ, Sol CM, Mulders A, Steegers EAP, Duijts L, Gaillard R, et al. Innovative approach for first-trimester fetal organ volume measurements using a Virtual Reality system: The Generation R Next Study. J Obstet Gynaecol Res. 2022;48(3):599-609.  365. Zhou X, Ji C, Sun L, Yin L, Deng X, Pan Q, et al. Clinical value of fetal facial profile markers during the first trimester. BMC Pregnancy Childbirth. 2022;22(1):738.  366. Altmann R, Rechberger T, Altmann C, Hirtler L, Scharnreitner I, Stelzl P, Enengl S. Development of the prosencephalic structures, ganglionic eminence, basal ganglia and thalamus at 11 + 3 to 13 + 6 gestational weeks on 3D transvaginal ultrasound including normative data. Brain Struct Funct. 2023;228(9):2089-101.  367. Liu F, Yang Z, Xi J, Yang L, Liu F, He H, et al. Ultrasound visualization of the central nervous system during embryonic and fetal periods: Neurosonoembryology utilizing multiple three-dimensional transvaginal ultrasound technology. Prenat Diagn. 2023;43(7):901-9.  368. Manzo L, Orlandi G, Gabrielli O, Toscano P, Di Lella E, Lettieri A, et al. Fetal Cerebellar Area: Ultrasound Reference Ranges at 13-39 Weeks of Gestation. Journal of Clinical Medicine. 2023;12(12).  369. Patabendige M, Kodithuwakku SU, Perera MNI, Dias TD. Fetal Echocardiographic Assessment: Impact of Gestational Age and Maternal Obesity. J SAFOG. 2023;15(4):377-81.  370. Schaefer EC, McKenna DS, Sonek JD. First trimester identification of fetal sex by ultrasound. 2023.  371. Tu KD, Oktay A, Aygun EG, Unsal G, Pata O. Evaluation fetal heart in the first and second trimester: Results and limitations. Niger J Clin Pract. 2023;26(6):787-94.  372. Weissbach T, Massarwa A, Hadi E, Lev S, Haimov A, Katorza E, et al. Early Fetal Corpus Callosum: Demonstrating Normal Growth and Detecting Pathologies in Early Pregnancy. AJNR Am J Neuroradiol. 2023;44(2):199-204. | | | |
